# Cloning, sequencing, and expression analysis of 32 NAC transcription factors (MdNAC) in apple

**DOI:** 10.7717/peerj.8249

**Published:** 2020-05-06

**Authors:** Huifeng Li, Kun Ran, Qinglong Dong, Qiang Zhao, Song Shi

**Affiliations:** 1 Shandong Institute of Pomology, Tai’an, China; 2 College of Horticulture, Northwest A and F University, Yangling, China; 3 College of Horticulture, Qingdao Agricultural University, Qingdao, China; 4 Nanjing Agricultural University, Nanjing, China

**Keywords:** Apple, MdNAC, Expression analysis, Gene cloning, Biotic stress, Abiotic stress, NAC transcription factor

## Abstract

**Background:**

NAC transcription factors play important roles in the regulation of plant growth, development, abiotic and biotic stress responses. The transcriptional level of *MdNAC*s in different tissues and under various biotic and abiotic stress treatments was determined to provide a solid foundation for studying the function of *MdNAC*s.

**Methods:**

Thirty-two full-length cDNA sequences of *Md NAC*s were isolated by homologous comparison and RT-PCR confirmation, and the obtained cDNA sequences and the deduced amino acid sequences were analyzed with bioinformatics methods. The prediction of subcellular locations of MdNAC proteins was performed using CELLO v.2.5, PSORT, and SoftBerry ProtComp 9.0. Expression levels of *MdNAC*s were detected in 16 different tissues using an array. Expression patterns of *MdNAC*s were detected in response to *Alternaria alternata* apple pathotype (AAAP) infection using RNA-seq, and the expression of *MdNAC*s was analyzed under NaCl and mannitol treatments using RT-qPCR.

**Results:**

The sequencing results produced 32 cDNAs (designated as * MdNAC24-39*, *MdNAC54-65,* and *MdNAC67-70* with GenBank accession No. MG099861–MG099876, MG099891–MG099902, and MG099904–MG099907, respectively). Phylogenetic analysis revealed that MdNAC34 belonged to the ATAF group, MdNAC63 belonged to the AtNAC3 group, MdNAC24, MdNAC26-30, MdNAC32-33, MdNAC35, MdNAC37-39, MdNAC56-57, MdNAC59-62, MdNAC64-65, and MdNAC67-70 belonged to the NAM group, and MdNAC25, MdNAC36, MdNAC54-55, and MdNAC58 belonged to the VND group. Predictions of subcellular localization showed that MdNAC24-27, MdNAC29-30, MdNAC33-37, MdNAC39, MdNAC54-65, and MdNAC67-70 proteins were located in the nucleus, MdNAC28 proteins were located in the cytoplasm, MdNAC31-32 proteins were located in the nucleus and cytoplasm, and MdNAC38 proteins were located in the nucleus and plasma membrane. Array results indicated that 32 *MdNACs* were expressed in all examined tissues at various expression levels. RNA-seq results showed that expression levels of *MdNAC26-28*, *MdNAC33-34*, *MdNAC60*, *MdNAC62-65,* and *MdNAC68* were induced, but *MdNAC24*, *MdNAC32,* and *MdNAC58* were down-regulated in response to AAAP infection. Under salt treatment, *MdNAC24*, *MdNAC27*, *MdNAC29*, *MdNAC34*, *MdNAC37*, *MdNAC39*, *MdNAC54*, *MdNAC59,* and *MdNAC63* transcription levels were induced. Under mannitol treatment, *MdNAC32* and *MdNAC54* transcription levels were induced, but *MdNAC24*, *MdNAC28*, *MdNAC30*, *MdNAC33*, *MdNAC35*, *MdNAC37*, *MdNAC55*, *MdNAC56*, *MdNAC58,* and *MdNAC59* were down-regulated. Taken together, the results indicated that the cloned *MdNAC* genes were expressed constitutively in all examined tissues. These genes were up-regulated or down-regulated in response to AAAP infection and to salt or mannitol, which suggested they may be involved in the regulation of growth, development, and stress response in apple.

## Introduction

The plant-specific NAC transcription factors (TFs) constitute a major TF family in plants, and play important roles in the growth, development, and responses to biotic and abiotic stress (Puranik et al., 2012). The name NAC refers to the first letter of three genes: N*AM* from *Pharbifis nil*, and A*TAF1/2* and C*UC2* from *Arabidopsis thaliana* (Aida et al., 1997). These genes are characterized by a conserved NAC domain of approximately 150 amino acids in length that is located at the N-terminal region. The NAC domain can be divided further into five subdomains: A, B, C, D, and E; A, C, and D subdomains are highly conserved, but the B and E subdomains are variable in different plants. The C-terminal sequence of the NAC TF is the transcriptional regulatory region, which determines transcriptional activation or inhibitory activity (Puranik et al., 2012).

NAC TFs are involved in the regulation of plant growth, development, and morphogenesis, which included seed germination (Kim et al., 2008), formation of lateral roots (He et al., 2005), formation of meristems and organ boundaries (Aida et al., 1997; Mao et al., 2007), secondary wall thickening (Mitsuda et al., 2007), floral organ development (Yoo et al., 2007), fruit maturation (Ríos et al., 2017), and plant senescence (Sperotto et al., 2009). The most in-depth and extensive research on the biological function of NAC TFs involves plant response to stress. Multiple NAC TFs are involved in plant abiotic stress responses to drought, high salinity, high and low temperatures, and waterlogging. NAC TFs, such as ATAF1, ANAC019, ANAC055, ANAC072 in *Arabidopsis*, and SNAC1, OsNAC5, SNAC2/OsNAC6, OsNAC10, and ONAC022 in *Oryza sativa* L., are involved in plant response to drought and high salt stress, and the functions of the corresponding mutants or overexpressed lines indicate that these genes play an important role in salt and drought resistance (Tran et al., 2004; Hu et al., 2006; Lu et al., 2007; Nakashima et al., 2007; Hu et al., 2008; Wu et al., 2009; Jeong et al., 2010; Takasaki et al., 2010; Hong et al., 2016).

NAC TFs were also involved in resistance to disease. NAC TFs positively or negatively regulated the resistance of rice and *Arabidopsis* to different types of diseases; rice *OsNAC6* and *Arabidopsis ANAC019* and *ANAC055* positively regulated the resistance of rice to blast disease (Nakashima et al., 2007) and *Arabidopsis* to *Botrytis cinerea* (Bu et al., 2008), respectively. Rice *RIM1* and *Arabidopsis ATAF1* and *ATAF2* negatively regulated the resistance of rice to dwarf virus (Yoshii et al., 2010) and *Arabidopsis* to *Fusarium oxysporum* (Delessert et al., 2005), respectively. In addition, transgenic *Arabidopsis* plants that overexpressed *Triticum aestivum TaNAC2*, *TaNAC67,* and *TaNAC29* increased tolerance to drought and high salinity (Mao et al., 2012; Mao et al., 2014; Huang et al., 2015). Transgenic plants of *Artemisia annua* L. and *Arabidopsis* that overexpressed *AaNAC1* increased drought resistance and resistance to *B. cinerea* (Lv et al., 2016a; Lv et al., 2016b), and transgenic *Ipomoea batatas* plants that overexpressed *IbNAC1* had greatly increased *sporamin* expression by binding the SWRE motif against mechanical wounding and herbivore attack (Chen et al., 2016a). Finally, transgenic *Solanum lycopersicum* plants that overexpressed *SlNAC4* and *SlJUB1* enhanced drought tolerance (Zhu et al., 2014a; Thirumalaikumar et al., 2018).

Although apple (*Malus* ×*domestica* Borkh.) is one of the most economically important fruit trees in the world, there have been few studies on NAC TFs in apple compared with the in-depth studies of NAC TFs in model plants like *Arabidopsis* and rice. Recent studies have shown that overexpression of apple NAC TFs *MdNAC029* and *MdNAC047* in transgenic apple calli and *Arabidopsis* increased tolerance to cold and salt stress, respectively (An et al., 2017; An et al., 2018). Overexpression of *MdNAC1* conferred the dwarf phenotype in transgenic apple (Jia et al., 2018). Through genome-wide analysis, a total of 180 *MdNAC* genes were identified in the apple genome that were clustered phylogenetically into six groups (I–VI) with the *NAC* genes from *Arabidopsis* and rice (Su et al., 2013). In view of the important role of NAC TFs in plant growth, development, and regulation of stress response, our study removed 23 *MdNAC* genes that already existed in the GenBank database through sequence alignment, cloned 32 *MdNAC* genes, analyzed their evolutionary relationships and subcellular localization of MdNAC, and studied the expression patterns in different tissues and organs under abiotic and biotic stress treatments. Our study provides a theoretical foundation for further study of the regulation of growth, development, and stress response by NAC TFs in apple.

## Materials & Methods

### Plant materials

The apple cultivar ‘Gala’ (*Malus* ×*domestica* cv. Gala) was used as material under stress conditions. *In vitro* seedlings of ‘Gala’ were cultivated on basic subculture medium (MS medium + 0.2 mg L^1^ indole-3-acetic acid (IAA) + 0.8 mg L^−1^ 6-benzylaminopurine (6-BA) + 30 g L^−1^ sucrose + 7g L^−1^ agrose) that was changed every 30 d. The cultivation conditions were under 14-h light/10-h dark and a temperature of 24 ± 2 °C. On the 20th day on the basic subculture medium, seedlings of similar growth and size (30 per treatment) were selected and transplanted to different media. The basic subculture medium was used as the control. We added 150 mmol L^−1^ NaCl or 300 mmol L^−1^ mannitol to the basic subculture medium to create different treatments (Li et al., 2019a; Li et al., 2019b).

### Gene cloning and sequence analysis

RNA was extracted in the fully expanded leaves of ‘Zihong Fuji’ apple (*Malus* ×*domestica* cv. Zihong Fuji) using the hot boric acid method, then cDNA was synthesized using a PrimeScript TM ^1ST^Strand cDNA Synthesis Kit (Takara, Dalian, China). Based on the nucleotide sequence of identified members in the MdNAC gene family, we designed primers for PCR (Table S1). PCR reaction conditions were 94 °C for 5 min, then 35 cycles for 94 °C for 1 min 20 s, 56–61 °C for 1 min 20 s, 72 °C for 3 min, and a final extension at 72 °C for 10 min. PCR products were purified and cloned into pMD19-T vectors to construct recombinant plasmids. The recombinant plasmids were transformed into the competent cells of *E. coli* DH5 *α*, and then the positive clones were selected.

The cDNA sequences that we obtained were used as queries in BLASTX searches against NCBI (https://www.ncbi.nlm.nih.gov/). Functional annotations (Gene Ontology) were analyzed with Blast2GO software (https://www.blast2go.com/). The open reading frame (ORF) and amino acid sequences were analyzed by DNAMAN 6.0, and a phylogenetic tree was constructed using MEGA 6 software, according to the NJ method (execution parameters: poission correction, pairwise deletion, and bootstrap (1000 replicates)). The conserved domains were predicted by Pfam 26.0 (http://pfam.xfam.org/) and the Conserved Domains program in NCBI (https://www.ncbi.nlm.nih.gov/Structure/cdd/wrpsb.cgi). CELLO v.2.5 (http://cello.life.nctu.edu.tw/), PSORT (https://psort.hgc.jp/form.html), and SoftBerry ProtComp 9.0 (http://linux1.softberry.com/) were used to predict subcellular locations (Dong et al., 2018a; Dong et al., 2018b; Dong et al., 2018c; Hao & Qiao, 2018).

### Expression analysis of *MdNAC* genes

Expression data for the *MdNAC* gene family in different tissues were obtained from Gene Expression Omnibus (GEO, https://www.ncbi.nlm.nih.gov/geo/) in NCBI (https://www.ncbi.nlm.nih.gov/), with GEO accession No. GSE42873 (Celton et al., 2014; Cui et al., 2015; Chen et al., 2016b; Zhang et al., 2016; Chen et al., 2017; Fan et al., 2017; Chen et al., 2018; Liu et al., 2018; Zuo et al., 2018; Li et al., 2019a; Li et al., 2019b; Zuo et al., 2019). These data included a set of expression arrays from 16 different apple tissues, with two biological replicates for each tissue, and an array probe was used as the MDP identification number in apple genome database V1.0. The RNA-seq data for *MdNAC* response to AAAP was from (Zhu et al., 2017). The treatment method was as follows: 2-year-old “Starking Delicious” cultivated apple plants grafted on *Malus robusta* rootstock were used as treatment materials. The fourth and fifth youngest opened leaves were collected from above plants and were inoculated with *Alternaria alternata* apple pathotype (AAAP) fungus. The AAAP fungus were grown on PDA medium for 5 days at 26 °C under dark conditions. In each treatment, leaves were inoculated with six cakes of mycelium applied to both side of the midrib of the abaxial leaf surfaces; the mock-inoculation of leaves using PDA medium cakes instead of mycelia was also carried out as a control. Thus, five groups of leaves were inoculated with mycelia or PDA cakes at 36, 18, 6, and 4 h intervals and then incubated at 25 °C under a 14 h light/10 h dark cycle in sterilized plastic chambers. The groups were then sampled simultaneously when the first set of inoculated leaves reached the 72 h post inoculation (HPI) time point; these five groups represent the five infection stage, 72, 36, 18, 12, and 8 HPI (Zhu et al., 2017).The RT-qPCR primers (Table S1) were designed based on the 3′- or 5′-UTR of *MdNAC* genes, and then RT-qPCR was conducted using a 3-step method by BIO-RAD IQ5 (USA) with *MdMDH RNA* as the internal reference gene (Perini et al., 2014). All RT-qPCR reactions were repeated three times. Each 20  µL RT-qPCR reaction mixture consisted of SYBR Green Master I 10 µL, 5 µmol L^−1^ forward prime 1 µL, 5 µmol L^−1^ reverse prime 1 µL, template 1 µL, and ddH_2_O 7 µL. RT-qPCR conditions were 95 °C for 3 min, then 40 cycles for 95 °C for 10 s, 58.5 °C for 30 s, 72 °C for 15 sand, after annealing to 55 °C, the temperature was increased 0.5 °C every 7 s till 95 °C, with 81 cycles in total. The 2^−ΔΔ*CT*^ method was used to analyze the data (Livak & Schmittgen, 2001).

## Results

### Cloning of *MdNAC* genes

**Figure 1:**
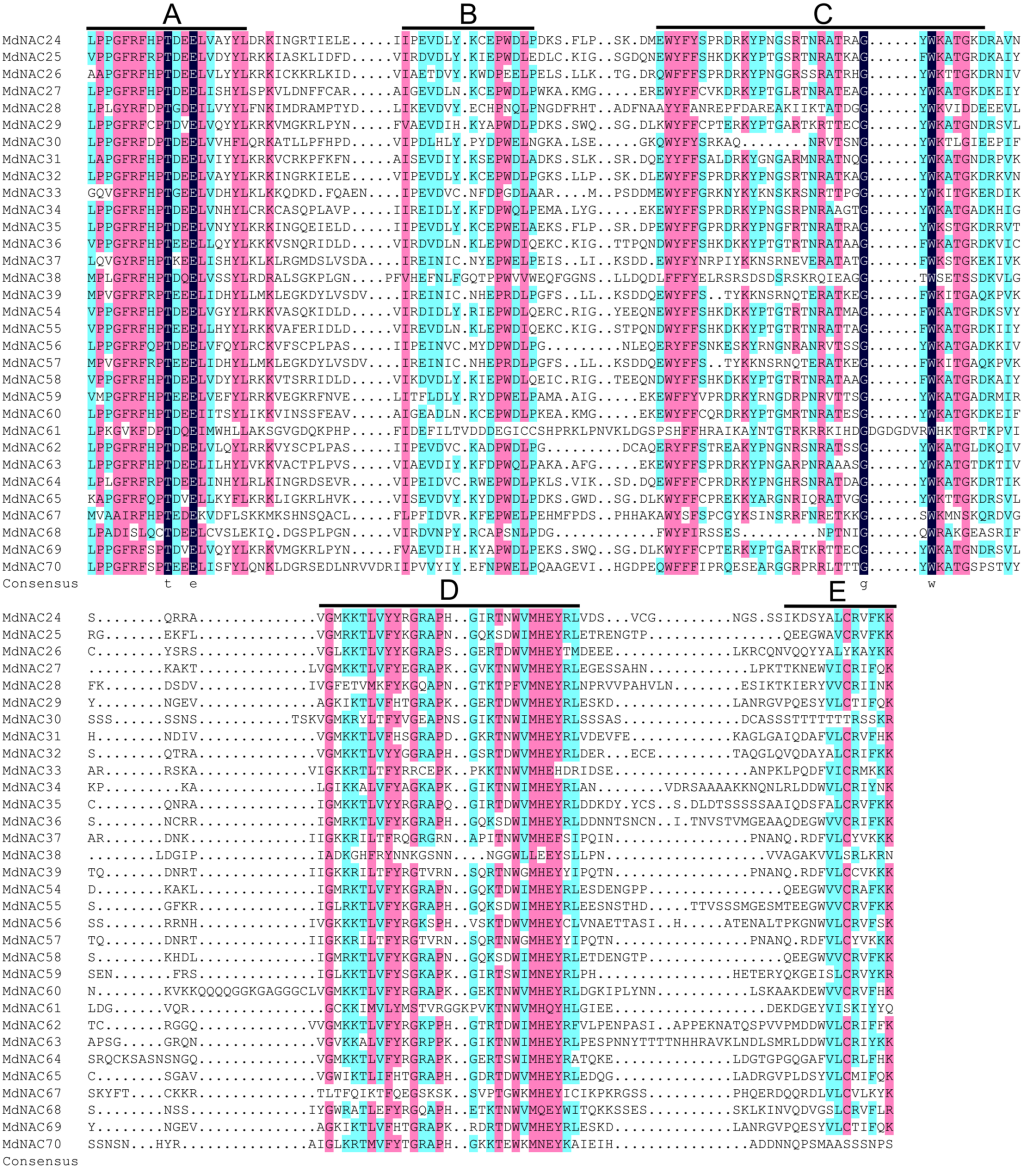
Sequence analysis of the NAC conserved domain in apple NAC proteins. Sequence alignment was generated by DNAMAN 6.0 software. The locations of A, B, C, D and E subdomains are indicated on top of the sequences.

A total of 32 *MdNAC* genes were cloned. Their gene names, gene IDs, GenBank accessions, genomic positions, lengths, molecular weights, isoelectric point (pI)-values, and information about Gene Ontology (GO) annotations were summarized for these MdNAC proteins (Table 1; Table S2). Homology alignment for the amino acid information showed that all MdNAC proteins contained a NAC conserved domain, and the NAC conserved domains could be divided further into A, B, C, D, and E subdomains (Fig. 1).

**Table 1:** The *MdNAC* genes in apple. (a) V1.0 gene ID represents gene ID from apple V1.0 database (Velasco et al., 2010) (b) GDDH13 gene ID represents gene ID from apple GDDH13 v1.1 database.

Gene name	V1.0 gene ID^a^	GDDH13 gene ID^b^	GenBank accession	GDDH13 Chromosome location	ORF	Amino acid	MW	pI
*MdNAC24*	MDP0000204582	MD10G1217500	MG099861	Chr10: 31583926-31588221	1,056	351	39.736	4.645
*MdNAC25*	MDP0000221977	MD00G1117000	MG099862	Chr00: 24815722-24817459	1,119	372	43.189	7.021
*MdNAC26*	MDP0000852271	MD05G1037700	MG099863	Chr05: 6012065-6015907	1,728	575	64.63	4.692
*MdNAC27*	MDP0000276765	MD17G1051600	MG099864	Chr17: 4033937-4035671	1,056	351	39.929	6.397
*MdNAC28*	MDP0000655623	MD16G1062600	MG099865	Chr16: 4451167-4452234	696	231	26.122	4.722
*MdNAC29*	MDP0000238035	MD10G1030400	MG099866	Chr10: 4011026-4014329	1,167	388	43.005	4.953
*MdNAC30*	MDP0000240094	MD08G1222300	MG099867	Chr08: 28493191-28494535	621	206	23.557	4.714
*MdNAC31*	MDP0000258167	MD07G1073100	MG099868	Chr07: 6943268-6950293	1,965	654	70.762	5.835
*MdNAC32*	MDP0000276982	MD09G1279900	MG099869	Chr09: 35666413-35671178	2,088	695	79.241	5.678
*MdNAC33*	MDP0000231845	MD07G1163200	MG099870	Chr07: 23835271-23837822	1,071	356	40.353	5.01
*MdNAC34*	MDP0000334047	MD15G1136600	MG099871	Chr15: 10025017-10026934	924	307	35.461	6.61
*MdNAC35*	MDP0000387787	MD14G1243700	MG099872	Chr14: 32218641-32221015	783	260	29.841	5.066
*MdNAC36*	MDP0000563165	MD07G1158300	MG099873	Chr07: 23095230-23097200	1,269	422	48.159	6.202
*MdNAC37*	MDP0000575835	MD07G1164000	MG099874	Chr07: 23874863-23877677	1,590	529	59.787	6.537
*MdNAC38*	MDP0000266267	None	MG099875	None	1,134	377	42.459	5.253
*MdNAC39*	MDP0000852815	MD01G1095100	MG099876	Chr01: 21019852-21023573	1,668	555	63.297	5.342
*MdNAC54*	MDP0000176447	MD15G1247800	MG099891	Chr15: 20579332-20584307	1,038	345	39.816	4.713
*MdNAC55*	MDP0000140229	MD14G1137900	MG099892	Chr14: 22516958-22518874	1,206	401	46.146	6.191
*MdNAC56*	MDP0000130686	MD06G1135700	MG099893	Chr06: 28061855-28063088	681	226	25.56	9.08
*MdNAC57*	MDP0000282616	MD07G1163700	MG099894	Chr07: 23860144-23863461	1,536	511	57.647	6.15
*MdNAC58*	MDP0000226701	MD11G1195600	MG099895	Chr11: 28030484-28032942	966	321	37.071	6.378
*MdNAC59*	MDP0000189394	MD08G1250000	MG099896	Chr08: 31430542-31432937	1,434	477	54.342	6.924
*MdNAC60*	MDP0000184069	MD14G1001200	MG099897	Chr14: 256059-258347	1,449	482	53.792	7.179
*MdNAC61*	MDP0000224592	MD13G1046600	MG099898	Chr13: 3226751-3230841	1,371	456	51.427	4.792
*MdNAC62*	MDP0000130123	MD16G1125800	MG099899	Chr16: 9127788-9129766	768	255	28.863	8.951
*MdNAC63*	MDP0000180683	MD14G1001000	MG099900	Chr14: 196247-198440	1,122	373	41.941	8.803
*MdNAC64*	MDP0000266908	MD00G1096600	MG099901	Chr00: 20298949-20303133	1,572	523	58.422	5.09
*MdNAC65*	MDP0000240855	MD04G1043700	MG099902	Chr04: 5142167-5148131	1,644	547	60.763	4.508
*MdNAC67*	MDP0000272823	MD01G1092800	MG099904	Chr01: 20755672-20758434	1,500	499	55.384	5.097
*MdNAC68*	MDP0000238773	MD15G1032200	MG099905	Chr15: 1983295-1985648	1,080	359	40.37	6.518
*MdNAC69*	MDP0000238035	MD05G1029800	MG099906	Chr05: 4849511-4852998	1,191	396	44.016	4.757
*MdNAC70*	MDP0000206212	MD12G1030200	MG099907	Chr12: 3262786-3264615	846	281	31.643	6.361

### Evolutionary analysis of MdNAC proteins

**Figure 2:**
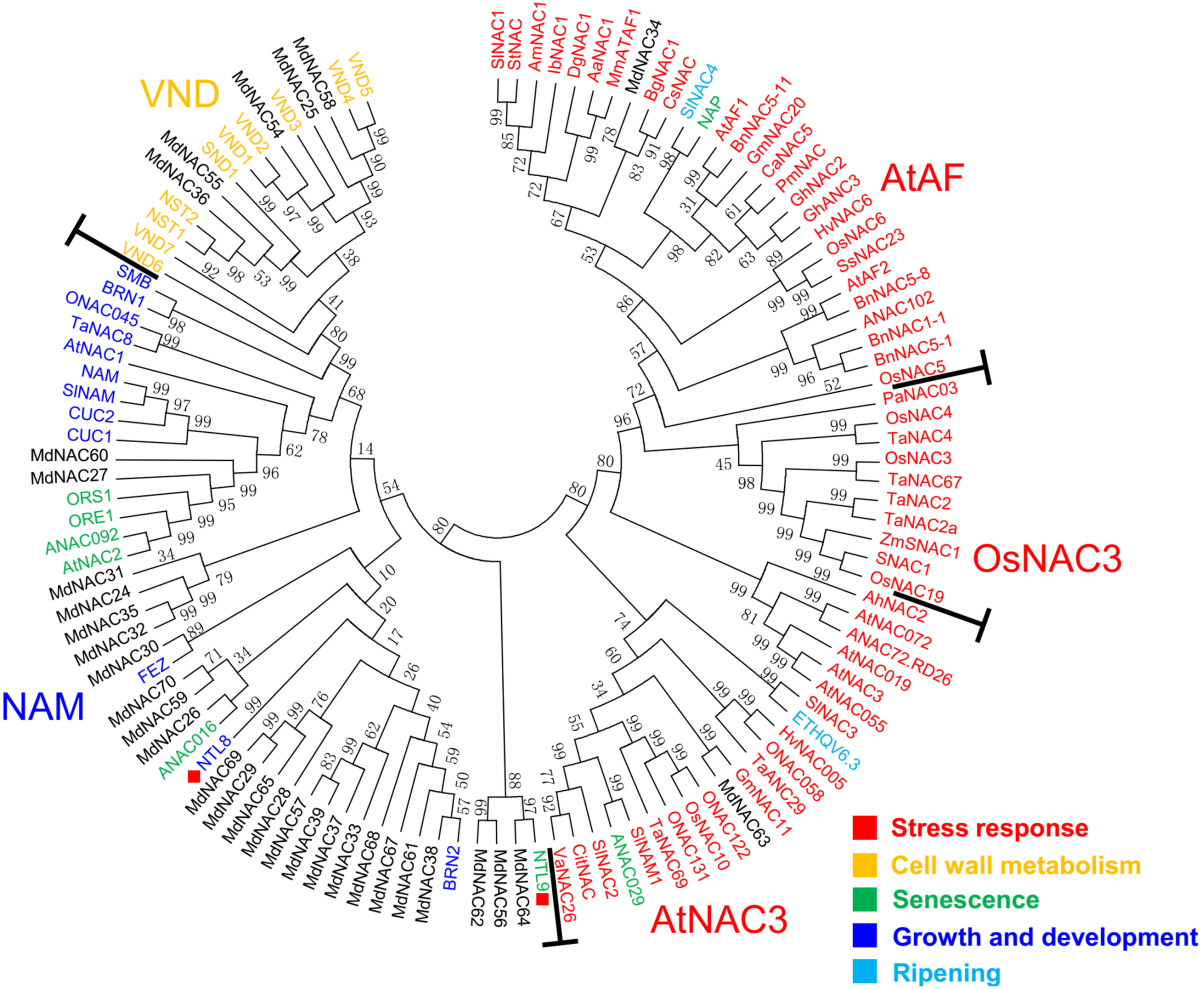
Phylogenetic relationships from NAC proteins of apple and known function of other plant species. Unrooted Neighbour Joining (NJ) phylogenetic tree was constructed with MEGA 6 software using full-length amino acid sequences from NAC proteins of apple and known function of other plant species. Colours indicate protein function: red, stress response; orange, cell wall metabolism; green, senescence; blue, plant growth and development; light blue, fruit ripening. Accession numbers and references of the NAC protein of each species are shown in File S2.

To understand the evolutionary relationships and potential biological functions among apple NAC proteins, cluster analysis of 32 apple NAC proteins and 86 other plant NAC proteins with known functions was performed using MEGA6 software. The result showed that 118 NAC proteins were divided into five subgroups: ATAF, OsNAC3, AtNAC3, NAM, and VND (Fig. 2). The NAC members of the ATAF, OsNAC3, and AtNAC3 subgroups mostly participated in stress response, the members of the NAM subgroup mostly regulated plant growth, development, and senescence, and the members of the VND subgroup were primarily involved in cell wall metabolism (Ooka et al., 2003; Zhu et al., 2014b). Further, proteins MdNAC34 and MdNAC63 were clustered into the ATAF and AtNAC3 subgroups, respectively; proteins MdNAC24, MdNAC26-30, MdNAC32-33, MdNAC35, MdNAC37-39, MdNAC56-57, MdNAC59-62, MdNAC64-65, and MdNAC67-70 were clustered into the NAM subgroup, and proteins MdNAC25, MdNAC36, MdNAC54-55, and MdNAC58 were clustered into the VND subgroup (Fig. 2).

### Analysis of subcellular localization of MdNAC proteins

Identification of subcellular locations of MdNAC proteins was performed using SoftBerry ProtComp 9.0. Predicted values for MdNAC24-27, MdNAC29-30, MdNAC33-37, MdNAC39, MdNAC54-65, and MdNAC67-70 to be located in the nucleus were the highest (a total of 10 points: higher value indicates greater credibility); the highest predictive value for MdNAC28 was in the cytoplasm, for MdNAC31-32 in the nucleus and cytoplasm, and for MdNAC38 in the nucleus and plasma membrane (Table 2). The subcellular localization of MdNAC proteins was analyzed further using online software CELLO and PORST. The prediction results showed that the localization of MdNAC proteins was consistent with the above results. All predictions indicated that MdNAC24-27, MdNAC29-30, MdNAC33-37, MdNAC39, MdNAC54-65, and MdNAC67-70 were located in the nucleus; MdNAC28, MdNAC31-32, and MdNAC38 were located in the cytoplasm, the nucleus and the cytoplasm, and the nucleus and plasma membrane, respectively.

**Table 2:** The information in predicting MdNAC subcellular localization.

Location	Nuclear	Plasma membrane	Extracellular	Cytoplasmic	Mitochondrial	Endoplasm. retic	Peroxisomal	Golgi	Chloroplast	Vacuolar
MdNAC24	9.99	0.01	0	0	0	0	0	0	0	0
MdNAC25	9.99	0	0	0	0	0	0	0.01	0	0
MdNAC26	6.59	0	0.7	0.33	0.63	0.6	0	0	1.16	0
MdNAC27	9.99	0	0	0	0	0	0	0	0	0
MdNAC28	0.61	0.51	0	6.47	2.42	0	0	0	0	0
MdNAC29	0.96	0	0	0	0.01	0	0	0	0.04	0
MdNAC30	9.99	0	0	0	0	0.01	0	0	0	0
MdNAC31	3.46	0.54	0	3.89	1.44	0	0.39	0	0.28	0
MdNAC32	3.36	0.65	0.36	3.28	1.04	0.13	0.2	0	0.62	0.36
MdNAC33	6.73	0.3	0.71	1.09	0.97	0	0.21	0	0	0
MdNAC34	10	0	0	0	0	0	0	0	0	0
MdNAC35	9.99	0	0	0	0	0	0	0	0	0
MdNAC36	9.99	0	0	0	0	0	0	0	0.01	0
MdNAC37	9.03	0	0.33	0	0.55	0	0.04	0	0.05	0
MdNAC38	4.09	4.98	0	0	0	0	0.3	0	0.45	0.19
MdNAC39	9.61	0	0.07	0	0.23	0	0.01	0	0.08	0
MdNAC54	9.99	0	0	0	0	0	0	0	0	0
MdNAC55	10	0	0	0	0	0	0	0	0	0
MdNAC56	9.95	0	0	0	0	0	0	0	0.05	0
MdNAC57	9.31	0	0	0.09	0.42	0	0	0	0.18	0
MdNAC58	10	0	0	0	0	0	0	0	0	0
MdNAC59	8.75	0.15	0.09	0.34	0.57	0	0.13	0	0	0.01
MdNAC60	10	0	0	0	0	0	0	0	0	0
MdNAC61	9.86	0	0	0	0	0	0	0	0.14	0.01
MdNAC62	9.98	0	0	0	0	0	0	0	0.02	0
MdNAC63	10	0	0	0	0	0	0	0	0	0
MdNAC64	4.31	0.97	0.32	2.25	1.46	0.3	0.08	0	0.21	0.1
MdNAC65	4.02	1.77	1.91	1.33	0	0.11	0.18	0	0.28	0.41
MdNAC67	6.66	0.57	0.69	0.59	0.97	0	0	0	0.53	0
MdNAC68	4.18	1.68	0.55	0.67	2.14	0.05	0.62		0.01	0.09
MdNAC69	9.98	0.01	0	0	0	0	0	0	0.02	0

### Expression analysis of *MdNAC* genes

**Figure 3:**
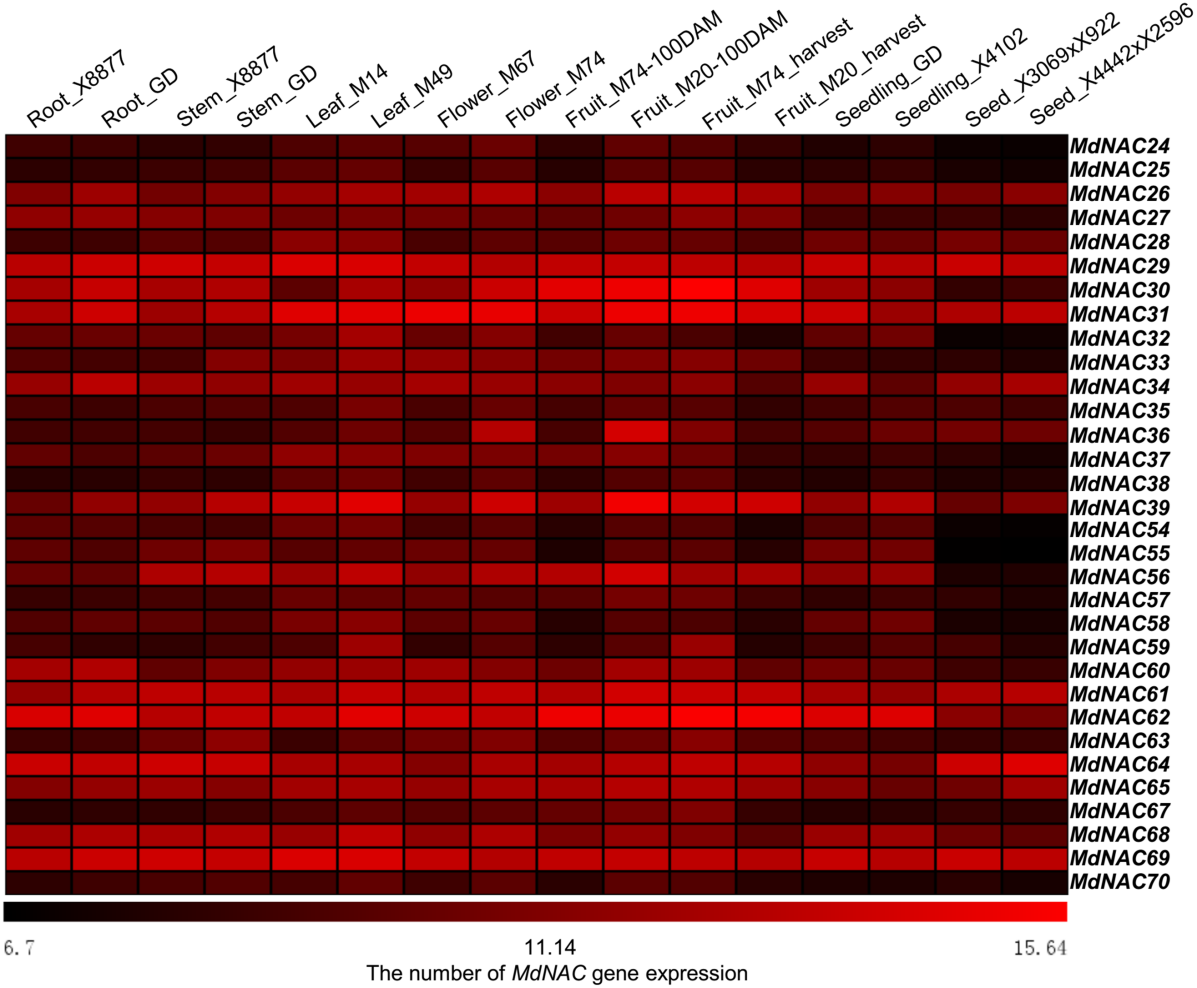
Expression profiles of apple *MdNAC* genes in various tissues. The data of apple *MdNAC* expression (GSE42873) in 16 different were searched at GEO database in NCBI. The heat map of apple *MdNAC* genes was generated by TIGR MeV v4.8.1 software. Each horizontal row represents a number of gene expression with its MdNAC, and the vertical columns represent different tissues from left to right.

The array (GSE42873) in 16 different apple tissues in GEO (https://www.ncbi.nlm.nih.gov/geo/) was used to evaluate the expression level of MdNACs in different tissues (Fig. 3). Thirty two *MdNAC* genes were expressed at different levels in 16 tissues of apple, and expression levels of *MdNAC29*, *MdNAC31*, *MdNAC61*, *MdNAC64,* and *MdNAC69* were relatively higher in all tested tissues, levels of *MdNAC26*, *MdNAC30*, *MdNAC39*, *MdNAC56*, *MdNAC62*, *MdNAC65,* and *MdNAC68* were relatively higher in multiple examined tissues, and levels of *MdNAC24*, *MdANC25*, *MdNAC35*, *MdNAC54*, *MdNAC55*, *MdNAC57*, *MdNAC67,* and *MdNAC70* were relatively lower in the tested tissues (Fig. 3).

**Figure 4:**
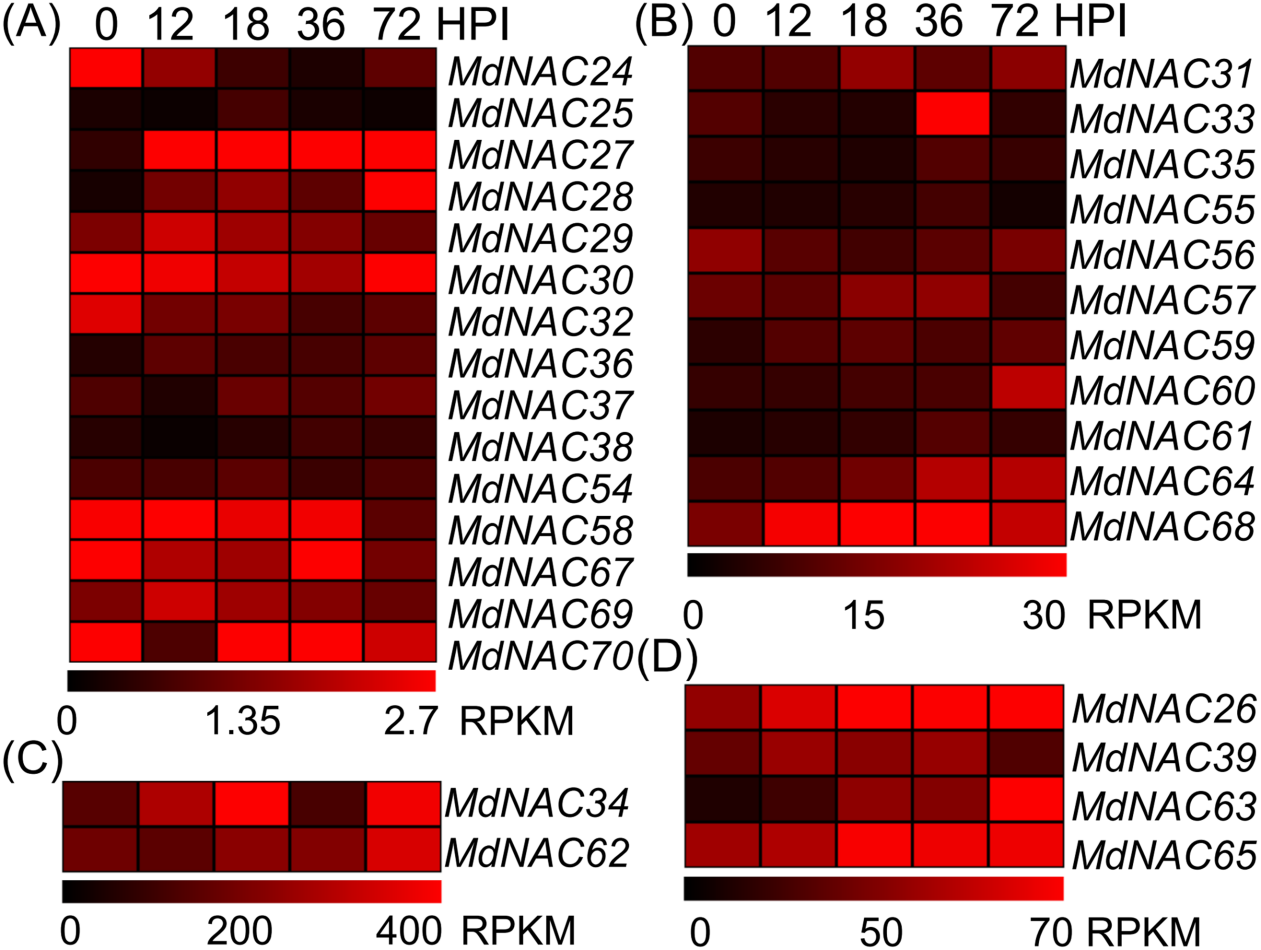
Expression profiles of apple *MdNAC* genes in response to *Alternaria alternata* apple pathotype infection. According to the RPKM values in RNA-seq, the expression data of apple *MdNAC* genes in response to AAAP infection were divided into four groups (A, B, C and D).The expression data of apple *MdNAC* genes in response to AAAP infection were obtained from supplementary data of previously published study *(Zhu et al., 2017)*. The heat map of apple *MdNAC* genes was generated by TIGR MeV v4.8.1 software. Each horizontal row represents a RPFM with its MdNAC, and the vertical columns represent 0, 12, 18, 36, and 72 from left to right.

Further, we detected the expression level of the response of *MdNAC* genes to AAAP infection. Expression levels of *MdNAC26-28*, *MdNAC33-34*, *MdNAC60*, *MdNAC62-65,* and *MdNAC68* were all up-regulated in the response of apples’ AAAP infection (Fig. 4). Particularly, the expression levels of *MdNAC27*, *MdNAC28,* and *MdNAC63* increased significantly, which was 17.2, 15.9, and 10.5-folds by 72 h post inoculation (HPI), respectively. Expression levels of *MdNAC24*, *MdNAC32,* and *MdNAC58* decreased, and the relative expression for the other *MdNAC* genes did not change significantly (Fig. 4).

**Figure 5:**
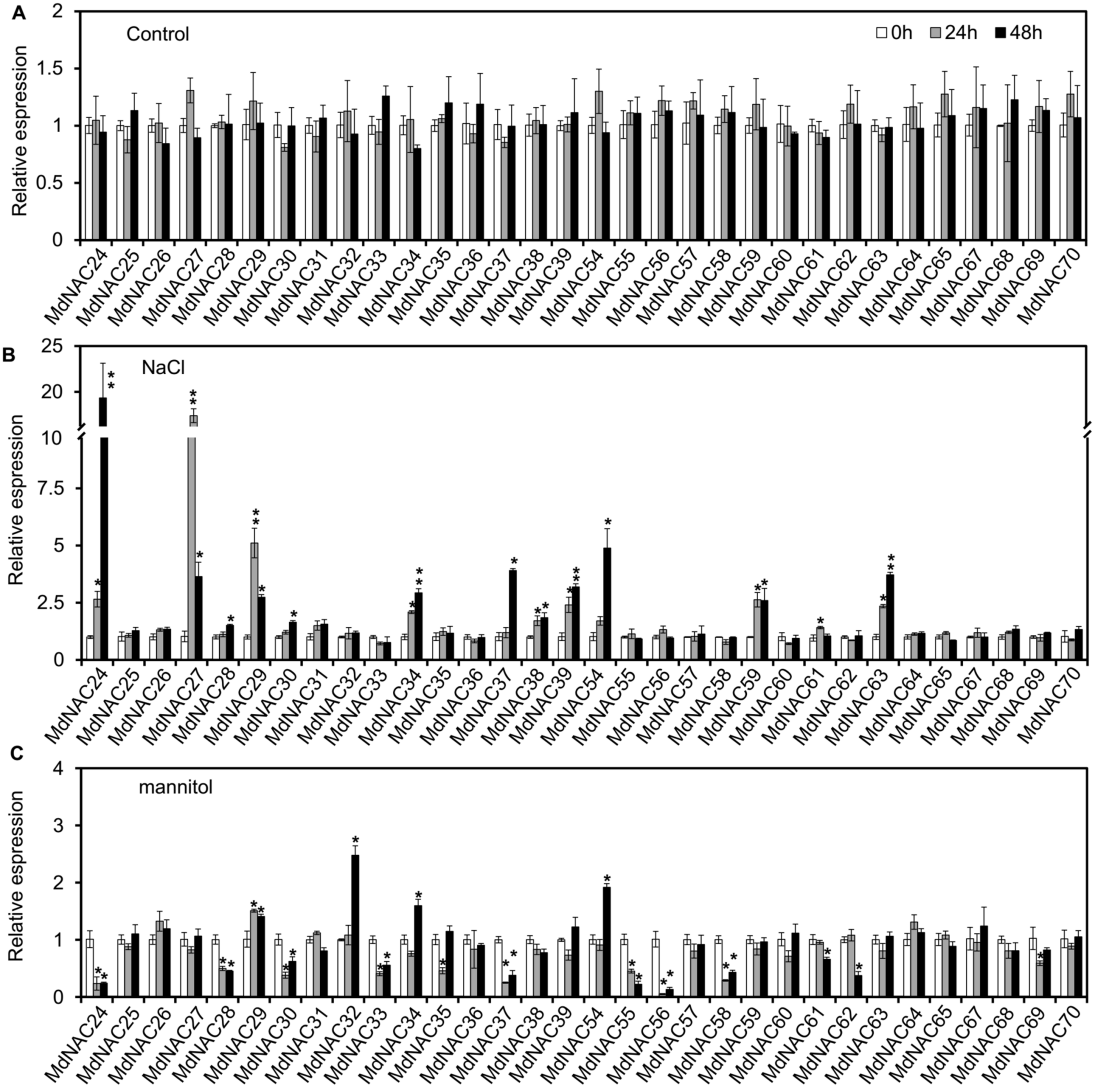
Expression analysis of apple *MdNAC* genes under normal growth, salt and mannitol treatments. (A) Expression analysis of apple *MdNAC* genes under normal growth; (B) expression analysis of apple *MdNAC* genes under salt treatment; (C) expression analysis of apple *MdNAC* genes under mannitol treatment. The reference gene used in qRT-PCR was *MdMDH*. Three independent biological replicates were used for calculations. Error bars indicate standard deviation. ^∗^ and ^∗∗^ indicate statistically significant differences, as determined by Student’s *t* tests, at *p* < 0.05 and *p* < 0.01, respectively.

Expression levels of *MdNAC* genes in ‘Gala’ seedlings under mannitol and NaCl stress were analyzed by RT-qPCR. Under mannitol treatment, 10 *MdNAC* members were down-regulated, which included *MdNAC24*, *MdNAC28*, *MdNAC30*, *MdNAC33*, *MdNAC35*, *MdNAC37*, *MdNAC55*, *MdNAC56*, *MdNAC58,* and *MdNAC59*. Among them, expression of *MdNAC56* decreased only 0.05-fold when treated for 24 h compared with that of the control (Fig. 5). However, expression levels of *MdNAC32* and *MdNAC54* increased 2.47 and 1.91-folds when treated for 48 h compared with that of the control, respectively. Relative expression levels of other *MdNAC* genes did not change significantly under mannitol treatment (Fig. 5). Under NaCl treatment, expression levels of *MdNAC24*, *MdNAC27*, *MdNAC29*, *MdNAC34*, *MdNAC37*, *MdNAC39*, *MdNAC54*, *MdNAC59,* and *MdNAC63* were up-regulated compared with that of the control. Among them, relative expression levels of *MdNAC27* and *MdNAC24* increased by 12.28 and 19.33-folds when treated for 24 h and 48 h, respectively, and relative expression levels of other *MdNAC* genes were not changed significantly (Fig. 5).

## Discussion

Based on the highly conserved domain in NAC TFs of plants and the draft genome sequence of the domesticated apple, 180 *MdNAC* genes were identified in the apple genome database v 1.0 (Velasco et al., 2010; Su et al., 2013). However, compared with other species, there has been little research on NAC transcription factors in apple. In this study, we cloned 32 *MdNAC* genes and detected their expression patterns in tissues and organs and their response patterns to abiotic and biotic stress. The multiple sequence alignment analysis showed that the NAC domain of the cloned MdNAC transcription factors in this study was highly conserved, and the C-terminus of their transcriptional regulatory domains was highly variable (Fig. 1). This suggested that MdNAC transcription factors played different important roles in regulating the growth, development, and response to abiotic and biotic stress in apple.

NAC transcription factors are one of the largest transcription factor families in plants; they have a large number of members, which determines their complex classification. Using different species of NAC proteins for cluster analysis and different software or algorithms may cause some differences in the results of evolutionary analysis of NAC transcription factors (Ooka et al., 2003; Rushton et al., 2008; Lv et al., 2016a; Lv et al., 2016b). The cluster analysis of 105 and 75 NAC transcription factors in *Arabidopsis* and rice, respectively, showed that NAC transcription factors could be divided into two groups: group I and group II, based on the sequence characteristics of NAC domains. Group I could be divided further into 14 subgroups, and group II could be divided into four subgroups (Ooka et al., 2003). Rushton et al. (2008) studied the evolution of 450 NAC proteins in *Arabidopsis*, rice, tobacco, poplar, and solanaceous plants, and divided the NAC proteins into seven subfamilies, of which six subfamilies were shared by all plants, and the other subfamily was unique to Solanaceae. The NAC proteins identified from poplar and watermelon genomes were clustered with NAC proteins from *Arabidopsis* and rice, and similar results were obtained; these NAC proteins could be divided into 18 groups (Hu, Qi & Kong, 2010; Lv et al., 2016a; Lv et al., 2016b). In addition, NAC transcription factors that were clustered in the same subgroup had similar functions (Fang et al., 2008; Su et al., 2013). For example, cluster analysis of the rice NAC families revealed that the NAC proteins associated with stress response belonged to class III, and the NAC proteins that were related to growth and development belonged to subclasses I-2, I-3, and I-4 (Fang et al., 2008). In this study, based on the results from previous studies (Ooka et al., 2003; Fang et al., 2008; Rushton et al., 2008; Zhu et al., 2014b), we conducted an evolutionary analysis of the NAC transcriptional factors that were reported to be related to plant growth, development, and stress response that included 32 MdNAC proteins (Fig. 2). The results showed that most members in groups ATAF, AtNAC3, and OsNAC3 were involved in stress response. Moreover, MdNAC34 and MdNAC63 belonged to ATAF and AtNAC3 groups, respectively, which suggested that the two MdNAC members were involved in stress response (Tran et al., 2004; Hu et al., 2006; Lu et al., 2007; Nakashima et al., 2007; Hu et al., 2008; Wu et al., 2009; Jeong et al., 2010; Takasaki et al., 2010; Hong et al., 2016; Lv et al., 2016a; Lv et al., 2016b; Chen et al., 2016a). The transcriptional levels of *MdNAC34* and *MdNAC63* were up-regulated under the treatment of AAAP and NaCl stress (Figs. 4 and 5), which indicated that *MdNAC34* and *MdNAC63* play important regulatory roles in response to biotic or abiotic stress, but further experimental verification is needed.

The results of phylogenetic analysis and functional verification of multiple NAC genes showed that NAC members in the NAM and VND groups were mostly related to plant growth and development (He et al., 2005; Mitsuda et al., 2007; Yoo et al., 2007; Kim et al., 2008; Sperotto et al., 2009; Ríos et al., 2017). In this study, we also cloned multiple *MdNAC* genes that belonged to the NAM and VND groups. Although previous studies have shown that these two groups of NAC members are mostly involved in plant growth, development, and morphogenesis. In this study, after biotic and abiotic stress treatments, the transcriptional levels of multiple *MdNAC* members in NAM and VND groups exhibited various expression characteristics (Figs. 4 and 5). For example, the transcriptional level of *MdNAC24* in the NAM group was up-regulated under NaCl treatment, but down-regulated by AAAP infection and mannitol treatment. The transcription level of *MdNAC32* in the NAM group was induced by mannitol treatment, but down-regulated by AAAP infection. The transcription level of *MdNAC32* in the NAM group was induced under NaCl treatment, but down-regulated under mannitol treatment. The transcription level of *MdNAC54* in the VND group was induced under NaCl and mannitol treatment, but exhibited no response to AAAP infection. These results suggested that the members of the NAM and VND groups participated not only in growth and development processes, but also in the plant’s response to environmental stress.

Recent studies have shown that several NAC TFs are membrane-bound transcription factors (MTFs), which are involved in plant growth, development, and various stress responses (Seo, Kim & Park, 2008; Seo, 2014; Yao, Deng & Zeng, 2017). For instance, overexpression of a TM-deleted, truncated a *Arabidopsis NAC* gene *NTL8 (NTM1-like 8)* form exhibit delayed flowering by suppressing the transcript level of *FLOWERING LOCUS T ( FT )* (Kim et al., 2008; Seo, Kim & Park, 2008). The transcript level of *NTL8* was triggered by high salinity *and GA biosynthetic inhibitor paclabutrazol (PAC)*, and was repressed by GA. *ntl8-1* mutants were insensitive to high salinity and PCA, indicating a role in GA regulation of salt signaling in seed germination (Kim et al., 2008; Seo, Kim & Park, 2008). The expression level of another *Arabidopsis* NAC gene *NTL9 (NTM1-like 9)* was induced by osmotic stress. Some senescence-associated genes were down-regulated in NTL9 knockout mutant but up-regulated in transgenic plants overexpressing a TM-deleted, transcriptionally active NTL9 form, indicating that NTL9 mediates osmotic stress responses during leaf senescence (Seo, Kim & Park, 2008; Yoon et al., 2008) . In this study, MdNAC38, NTL8, and NTL9 belonged to the NAM subgroup, MdNAC38 was predicted to be located in the nucleus and plasma membrane, and was slightly induced by salt stress, indicating MdNAC38 as MTF may be involved in salt response. However, further subcellular localization and functional analyses are needed to analysis this possible molecular mechanism.

## Conclusions

Thirty-two novel *MdNAC* genes have been successfully isolated from *Malus domestica*, belonging to subgroup ATAF, OsNAC3, AtNAC3, NAM, and VND of this plant transcription factor family. Array, RNA-seq, and qRT-PCR-based transcription profiling indicated that 32 *MdNAC* genes were expressed in all examined tissues at different expression levels, and responded differentially to various stresses, suggesting that these genes may be involved in the regulation of growth, development, and stress responses in apple. These results serve as the theoretical basis for understanding the biological function and regulation of apple NAC transcription factors.

##  Supplemental Information

10.7717/peerj.8249/supp-1 Table S1 Application of primers and sequences

10.7717/peerj.8249/supp-2 Table S2 Functional annotation (Gene Ontology) of MdNAC proteins

10.7717/peerj.8249/supp-3 File S1 cDNA sequences of 32 MdNAC TFs

10.7717/peerj.8249/supp-4 File S2 Protein sequence accession numbers and references for each species

10.7717/peerj.8249/supp-5 File S3 The expression data of apple *AP2/ERF* genes under normal growth, mannitol and salt treatments

10.7717/peerj.8249/supp-6 File S4 Homology comparison of the deduced amino acid sequence alignment of MdNACs

## Additional Information and Declarations

### DNA Deposition

The following information was supplied regarding the deposition of DNA sequences: The MdNAC24-39 sequences are available at GenBank: MG099861 to MG099876. The MdNAC54-70 sequences are available at GenBank: MG099891 to MG099907.

### Data Availability

The following information was supplied regarding data availability: The expression level of *MdNACs* in different tissues is available at NCBI GEO: GSE42873. The expression level of the response of *MdNAC* genes to AAAP infection is available at https://www.frontiersin.org/articles/10.3389/fpls.2017.00022/full.
