# Supplementary material for: Cloning, sequencing, and expression analysis of 32 NAC transcription factors (MdNAC) in apple
Source: PeerJ. 2020 May 6;8:e8249. doi: 10.7717/peerj.8249 (PMC7210808; doi:10.7717/peerj.8249)
Supplement: Table S1 [file peerj-08-8249-s001.docx]

| Use | Primer name | Forward Primer Sequence (5'- 3') | Reverse Primer Sequence (5'- 3') |
| --- | --- | --- | --- |
| Complete | *MdNAC24* | ATGGCACCCATGAGTCTTCCTCCT | TTAGTCTTGCATGGAGAGGGCCAC |
| ORF | *MdNAC25* | ATGAATACGCTTTCATCACATGTA | TCATTTCCAGAGATCAATTTGGAA |
| amplification | *MdNAC26* | ATGGTGACGGAGGACGATCCAGCT | TCAAGCGGGGAGGCATCTCCCCGG |
|  | *MdNAC27* | ATGGAAAATACTTCTGGGTTTATT | TCAATAATACCAGAGGCCATCGAG |
|  | *MdNAC28* | ATGTCTTCCTCCATGGCATCCTCA | TCACTTTGGTACTCCATCTTCAAC |
|  | *MdNAC29* | ATGGGGAAAGGGAAATCATCGTTG | TCAACTGGTCTCACCCTGAAAAGG |
|  | *MdNAC30* | ATGGGAGATAACCAGTTTAAGCTC | TTAATTTGGCAAACTTATTTCATC |
|  | *MdNAC31* | ATGGGTCGAGAAGCGGACCTTCAG | TCAGACCTTCTTCTTCAAATCCTC |
|  | *MdNAC32* | ATGGCTCCTGTTTCATTGCCTCCT | TCAAGAGGGAATTACATTGTGGTT |
|  | *MdNAC33* | ATGGAAACACCGGGAAGAAAGAGA | CTACAGTGTGTAGTCCTCTAGTTG |
|  | *MdNAC34* | ATGAGCGGAGGAGGAGACGAGCAG | TTAAAATTGGGTGTGGAGATAGGA |
|  | *MdNAC35* | ATGGCGCCAGTGGGATTACCTCCC | TTAGTTTGTGAAGGCCACATGAGA |
|  | *MdNAC36* | ATGGCGCCTGAAAACATGAGTATA | TTATATAGGACCGGTCGACACGTG |
|  | *MdNAC37* | ATGGCTTCCAATGGAATCCGCTTC | CTAGCACCACTGCCCGTATAAACC |
|  | *MdNAC38* | ATGGAGGTGGGTTATTACGGTATG | CTATTGATCATTCACAGAATCGTT |
|  | *MdNAC39* | ATGGCTGCCAATAGAAACCGCTTC | CTAGCACCACTGCCCGTATAAAACT |
|  | *MdNAC54* | ATGATGGAGTCAATCGAGTCTACT | TTATTTTTCAAATATGCATATCCC |
|  | *MdNAC55* | ATGTCTGATGATCATATGAGTCTA | TTACACCGACAAGTGGCAAAGCGG |
|  | *MdNAC56* | ATGGACAAGTTCAAATTTGTTAGA | TCAAAATTTAGGGCAGCTACTGAT |
|  | *MdNAC57* | ATGGCTGCCAATAGAAACCGCTTC | CTAGCACCACTGCCCGTATAAAAC |
|  | *MdNAC58* | ATGGAAACTTTTTCACACGTTCCC | TCATGAGTAGCTATTTCCCTTTGA |
|  | *MdNAC59* | ATGGCAGTTGCAGAAACAACAACA | TTACTTGAACAGATTGTTGTAGTC |
|  | *MdNAC60* | ATGGATCCGTCGATTGAAGCACAA | TCAAAAGCCCCACAAGCCTTCAAT |
|  | *MdNAC61* | ATGGCTGGGCCATCATGGTTGTTT | TCAGTCAGCCACCTTGCCACCCCA |
|  | *MdNAC62* | ATGGAGAGGATTAATTTTGTGAAG | CTACTGTTTTCTTCTAATTAAACC |
|  | *MdNAC63* | ATGAAAAACCCAGAATCAAGCCTG | TTATTCTTGAAAATGAAAACGAGG |
|  | *MdNAC64* | ATGGCGGAGCTGTCGATGGAATCG | CTAAAATTTAGGATCAAAGCCATG |
|  | *MdNAC65* | ATGGGGAAAATGTTTAAGGCTCCT | TTACACAGAATTATCGCCATTCAT |
|  | *MdNAC67* | ATGAGAAACATCAGCAGCAGCAGC | TCAAGAACTTTGTGGGAAAAGAGT |
|  | *MdNAC68* | ATGTATCCGCAATCAGCTGCCCTG | CTAAAACGGCATGAAGCAGAAATA |
|  | *MdNAC69* | ATGGGGAAAGGGAAATCATTGTTG | TCAGAAGTTCACGTTATCAAACAC |
|  | *MdNAC70* | ATGGAGGATTTACCACCTGGATTT | TTAAAGCAAAAAATTTATCATTTG |
| RT-qPCR | *MdNAC24* | ACAAACACAGCAAAGCCAGA | TAGTCTTGCATGGAGAGGGC |
|  | *MdNAC25* | ACATTGGACAGGACTATGCC | CCCTTTAAAGTACCGTACCTCC |
|  | *MdNAC26* | GCAGGCATAAGGAGGGGATT | AGCAGCAAAACTTCCCACTT |
|  | *MdNAC27* | TGCAGCACCAAAACTCAGTT | CATCAATGCTCAGCCCTGTG |
|  | *MdNAC28* | TGAGAGGTACGTGGTATGCC | GCTTCTGCCCCATCACTTTG |
|  | *MdNAC29* | TTTGGAGCTGGATGATCTTGG | GTCTCACCCTGAAAAGGCATC |
|  | *MdNAC30* | TGGGACAGAGCTTTCATGC | GCACTAGTTAGGTCCCTCTCT |
|  | *MdNAC31* | CAAGCCCGAATTGATCTCCT | AGATAGTTCAACGAGGCCCA |
|  | *MdNAC32* | TACAAGTGTGAGCCATGGGA | CGCCTTCCAGTATCCAGCTT |
|  | *MdNAC33* | TGTCCTCCACCAGAAGAAGA | TTCGTACCACTCCTGCACTT |
|  | *MdNAC34* | ACATGGATGGCTTCTCGGAT | TGTTCTCTCCGTCTTGCCAT |
|  | *MdNAC35* | CATCGTCAATCTCATGCCCG | GAAGGCCACATGAGAGAGCT |
|  | *MdNAC36* | GGACAAACCGAAGCCTCTAG | ATGGTGAGTGGGATGGTAGG |
|  | *MdNAC37* | CACGGTTCTAGGTGCGATAT | ACATGGCTGATACTCCCACA |
|  | *MdNAC38* | ATGTGATGCCCATTCCCACT | TAATGTCTCCACGCACTGCT |
|  | *MdNAC39* | CAGTGGTGCTAGAGGGGTT | CACTTCATCACTGCAAACCG |
|  | *MdNAC54* | TGTCATTGTTGTTGTTGCAGAG | CCCCAAGTGTTGTCCCTCTT |
|  | *MdNAC55* | TCCCATCATCAATCCGACGT | TGTCGTTTCAAGTGCATCCC |
|  | *MdNAC56* | AGCTTCCATTCACGCAACTG | GGAAGAAGATGCAGGATTCGC |
|  | *MdNAC57* | AGTTTTATACGGGCAGTGGTG | CACTTCATCACTGCAAACCG |
|  | *MdNAC58* | CGGCTGTCCTTGTTTCTTCC | CAGCTTTTGGTCTTCGGTCA |
|  | *MdNAC59* | AAATTTCCCTTCAGCGGCG | CAGATTGTTGTAGTCCGGCC |
|  | *MdNAC60* | GCACCGACATGAACACTACC | CCCCACAAGCCTTCAATGTC |
|  | *MdNAC61* | ACAAGCACATGTCAAACCCC | AGTCGGAAATCAGGAGGTGT |
|  | *MdNAC62* | TGACGAAAGACAGGGCAAAT | TGGAACTACTGCTTTCTTCGTG |
|  | *MdNAC63* | CCATCATCAGCCCCAAACAC | TGAATTGGCGGTGTTAGTGA |
|  | *MdNAC64* | AGAGGGATGATGGCTGGTAC | AACATCCCAGAGAACCCAGG |
|  | *MdNAC65* | ACAATTTCTCTGGCGTGCAT | ACCCATAGATCCCTTCCACT |
|  | *MdNAC67* | TTGAAGGAGAGGAGGAGGGA | TCTTCAGTGGGATGGAACCT |
|  | *MdNAC68* | CTCCGAATCCTGGCTCATCT | CCACCAACATTAGCATTCTGAGA |
|  | *MdNAC69* | CTGGATGATCTTGGCGAACC | ACACGTTCAACTGGTCTTCAC |
|  | *MdNAC70* | TAGAGGCAGGGAGATCGAGA | AGTCCTCACTCCTCCCATCT |
|  |  |  |  |
