# Supplementary material for: Cloning, sequencing, and expression analysis of 32 NAC transcription factors (MdNAC) in apple
Source: PeerJ. 2020 May 6;8:e8249. doi: 10.7717/peerj.8249 (PMC7210808; doi:10.7717/peerj.8249)
Supplement: File S1 [file peerj-08-8249-s003.docx]

>MdNAC24 [Leaf=Malus domestica] NAC domain class transcription factor

ATGGCACCCATGAGTCTTCCTCCTGGATTCAGATTCCACCCAACAGATGAAGAGCTTGTTGCTTACTATCTAGATCGAAA

AATCAATGGTCGCACCATTGAGCTAGAAATTATCCCAGAGGTCGACCTCTACAAATGTGAGCCATGGGATTTACCTGATA

AGTCGTTTCTTCCGAGCAAAGACATGGAGTGGTACTTCTACAGCCCGAGGGATAGGAAGTACCCCAACGGGTCGAGAACG

AATAGGGCTACTCGAGCTGGGTACTGGAAAGCGACTGGGAAAGACCGGGCGGTGAACAGTCAGAGGCGTGCTGTCGGCAT

GAAGAAGACATTGGTTTACTATAGAGGTAGAGCCCCCCATGGTATTAGAACCAACTGGGTTATGCATGAGTACCGGCTGG

TCGATTCTGTGTGTGGCAATGGGTCATCATCTATAAAGGATTCTTACGCATTGTGCCGAGTGTTCAAGAAAACAATACAA

ATGCCCAAGAGTAACAAAGAATACACGCCGATTGGGATTAACAATGCAGACAATGATTCAATATGGGTCTCCAATGAACA

ATTGTTAGGGGAAGACACTAGTGGCATTAATGAGGGAGCTTCAAGAGGGATTGAAACTGATCAAGATGAGAATGATTCCA

ACCATGATTATCCCAAATTTCTATCTGACACCTCTTCTTCAGATCTCACTCAAGGCACACCTACTGAAACTGGCATAGCT

GATGATTTACAAGCTCCATTTGCTTCTGATGAAGCAAACAGTTCAGCTGATCTATACTCTTTTGGTGTCCACTGCTCCTC

AGATCTACTTCAGGAAACATATATACCGCCCAATGCGAGCTCACTGAATACCTATCAATTTCCTTACCCACCCTTAGAAC

TTGAAGACTTCCCGCAGATCAATTTAGCTGCAGAGACAAACACAGCAAAGCCAGAAATCATCGACAAGTACATGTCGTAC

GACAAGTTCAAGGATTACATGAATGGATCATTTGAAGAGATCTTCTCTTTATGTTCATCTCAAGACAACTCTGTGGCCCT

CTCCATGCAAGACTAA

>MdNAC25 [Leaf=Malus domestica] NAC domain class transcription factor

ATGAATACGCTTTCATCACATGTACCCCCAGGCTTTCGATTCCGTCCCACAGATGAAGAACTTGTTGATTATTATCTCAG

AAAGAAGATTGCTTCGAAACTGATTGATTTTGATGTCATCAGAGATGTTGATCTTTATAAGATTGAGCCGTGGGATCTTG

AAGACTTGTGCAAGATAGGAAGTGGTGATCAGAATGAATGGTACTTCTTTAGCCACAAAGATAAGAAGTACCCTACTGGA

AGTCGTACAAATAGGGCAACGAAAGCCGGGTTTTGGAAAGCTACAGGAAGAGATAAGGCTATTTATAGAGGAGAAAAATT

CCTCGTTGGGATGAGAAAGACCTTAGTCTTTTACAAAGGCCGTGCTCCTAATGGACAGAAGTCAGATTGGATCATGCATG

AATATCGACTAGAAACTAGAGAAAATGGAACTCCTCAGGAAGAAGGATGGGCTGTGTGTAGGGTGTTCAAGAAAAAGTTG

GCAGCCGTGAGCAGAATGGGAGATTACGTGTCACCAGATTGCTATGATCAAGTCTCATTCCTGCCACAACTTGATAACCC

TCCAAGGACATCAATGTCCCGCACTTATGCATCGCAAAATGAGCAGCAGCTACACTATTCACAATGCAAGAAAGAGTTTG

ATTTGCAGTACAACATGCCACATCATCATCATGACTCTTTTCTCCAGCTCCCTCAATTGGAAAGCCCCAAAGTTCCTCAT

CAGTCAGCTACCCCATATGGTAATTGTGCTATGCAGTCTTCCACACTCACACAAGAGGAGCAATTTGCGCAATACATCTC

CCAGCAAAATATGTTCAAATCATCGTCATCGCCATCGGTACATGCGCTTGACAACAACAATGATTATCTGGAAGCTGTTG

ATCAAGTGAAGGATTGGCGAGCCCTTGACAAGTATGTTGCGTCACAGCTCAGCCATGACCAGCAAGATGCTTCCAAGAAA

GTAACAAATTACTCTCATGCAGAGGAAATATTTCATGTGGCTGAACACATTAATATGCTTGCCAATGACGCATCCAGAAG

GCGAGACAATAATAATAACATTGGACAGGACTATGCCTCAACATCAACCTCTACTTTCCAAATTGATCTCTGGAAATGAT

CAAGAACATGAATATTACAGTAACTATTAGCCAATACATAATACTAATTAATCGTAGGAGGTACGGTACTTTAAAGGGAT

CGATATTTGAGTATTATTAATCCAGTTGCTGTACATAAGAAAACACAATTATGGTATTATTTTTACCTATTCGACTTTAA

ATTTGTGACTTTTTGTGTTGATGAATATATACTAGGGCAACTAGGTGGACCAATT

>MdNAC26 [Leaf=Malus domestica] NAC domain class transcription factor

TCTCTCCTCTGCCCTAGGAGAGTCCTACGGTGATTATGTAGGACTTGTCTTCTCTCTTTCTATCCACATCGCTCTCTCTT

GCTTCTGATCGATTGATTTGTGCTTCAAGTTTTTCAATTTTTCTTTGTTTGAGGGTTTTTATTTTGTTGGAGAGGAAGCG

ATGGTGACGGAGGACGATCCAGCTGCGCTTTTCTGCGATGGACACTTTGCCGCGCCGGGGTTTCGGTTCCATCCCACCGA

CGAGGAGCTTGTCCTCTACTATCTAAAGCGGAAGATCTGTAAGAAGAGGCTCAAGATTGACGTCATCGCCGAGACCGACG

TCTACAAGTGGGACCCTGAGGAACTTCCCGAACTATCTTTGTTGAAAACTGGAGACAGGCAATGGTTCTTTTTCAGTCCT

AGAGATCGGAAATACCCTAATGGAGGAAGATCAAGCAGGGCAACCAGACACGGGTATTGGAAAACTACAGGAAAGGATCG

TAATATTGTATGTTATTCACGGTCTGTTGGATTGAAGAAGACCTTAGTTTATTACAAAGGCCGTGCACCTAGTGGGGAGC

GCACTGACTGGGTGATGCACGAGTATACCATGGATGAAGAAGAGCTCAAGAGATGCCAGAATGTACAGCAATATTATGCT

CTCTACAAGGCTTACAAAAAAAGTGGACCTGGTCCCAAAAATGGCGAGCAATATGGGGCGCCATTTAGGGAAGAAGACTG

GGTTGATGATGAATCCCCAGTAATCAAGACCTCTGCTGATTGCCAAATCTCAGTGAAGGAAGCTGTAGATGTTGTTTCTA

TTGATAATGTGAAAGGCAATGGCGAAGTCCATTATGCACTAGATGACATCGAGGAATTCATGAAACAGATGGCTGATGAG

GCTGTGCTGCCACAAATGAATGGGTACGGTTACACAGCACCCCAGGTTGTTAGCGAAGAAGATACCCAAAGTGGTCTGGT

TGATCTGTACTCCAGGGAAGTTTTCAGTCCAGAACTTATCAAAGAGTTCAATCCAAATGTGCAGCAATGCAATGTGGAGG

CCAACTTTGACTTCACACAGTCAGCTACCTCCCAAATGCAATCACATGAGGCATCTGAAGTCACCACAGCTGTTCCTGAC

ATTTATGAGCACGGTCCTCCTGCTCTGCATGAAGAGGATTTCTTGGAGATGGATGATCTCCTTGGTCCTGAACCTACTGT

ACCAAACACTCTGAATCCTGTGGATGACTTGCAGTTTGGAGAGCTAGATGGATTAAGTGAATTCGACTTGTATCATGATG

CAGCCATGTTTCTTCATGACTTGGGGCCTATTGATGAAGGAAATATTTCTCATCATCAGTACATGAATTCACAGGGGAAT

ACTATTGTAGACCAATTTGAATACCACTTACAACCTAATCCAGCAGCTGAAAACCAGGTTAACCATATGCTGAATCCAGA

ATTAGCTCAGATGAACAATCAGCTGTGGACAAACACTGAAAGAGCAGAACCGAACCAGGGATCTCTCTCGTATTCAACCT

CAGGTGTGGTATATGAATCTTCAAATTTTCCTTCCCGAGCAACTCAAAATCAAAGTGGCAATGAGGCTGCAGGTGCTACA

AGTCAGTTCTCTTCTGCTCTTTGGGCATTTGTGGAGTCTATACCTACCACTCCCGCATCTGCTTCAGAGAGTGCTTTGGT

GAATCGTGCTTTCGAACGGATGTCTAGCTTCAGTAGGTTGAGAATTAATACGGCAAGTACAACTGTCGCAGCAGGTAATG

GTTCTGAAACTATGAGAGCAGGCATAAGGAGGGGATTCTTCTTCCTTCCGGTTCTTGTAGCACTATGTGCCATTTTTTGG

GTTTTGATGGCAACTCTCAGACCGCCGGGGAGATGCCTCCCCGCTTGAGTCTGCAAGTGGGAAGTTTTGCTGCTATACAT

ATATATATAACATATAATTATTTATATTCATCGTCGGAAAGTTGATGGGAAGTGATTTCAAATCATTATGAAAACATAGG

ATTCTAATATCTATCTGAATTTTGGTTGTAGAGGATGAATTTTAGCTCCTTTTTCCTTTACAAAAGTAAATAGGAAATTT

AACATGAATTTTTCGGTCGCAAA

>MdNAC27 [Leaf=Malus domestica] NAC domain class transcription factor

ATGGAAAATACTTCTGGGTTTATTATGGAAGATGAGCAGATGGAACTTCCTCCGGGATTCCGATTTCATCCGACGGATGA

AGAGCTCATAAGTCACTACCTGTCCCCCAAAGTTCTTGACAACTTCTTCTGTGCAAGAGCAATTGGCGAGGTGGATTTGA

ACAAGTGTGAGCCTTGGGATTTGCCTTGGAAGGCAAAAATGGGAGAAAGGGAATGGTACTTCTTCTGTGTGAAAGACAGA

AAATACCCAACTGGTCTGAGAACAAACCGGGCAACGGAGGCCGGGTACTGGAAAGCCACAGGCAAAGATAAGGAGATTTA

CAAGGCCAAAACCCTGGTTGGAATGAAGAAAACTCTGGTTTTCTACGAAGGAAGAGCCCCAAAAGGTGTGAAGACCAATT

GGGTCATGCATGAATACAGATTGGAGGGCGAAAGCTCTGCCCATAATCTCCCCAAAACTACGAAGAATGAGTGGGTGATT

TGCAGAATTTTCCAAAAGAGTAGTGGTGGGAAGAAGACTCATATTTCAGGGTTGCTGCGGCCGAGCCCCTTCGGGAACGA

ATTGCGCTCTTCTTTACTCCCGCCATTAATGGATTCTCCAGCCTACAACAGCGACGCTAGAACAACAACCACCGCCTGCG

AAACCTCTCACGTGTCCTGCTTTTCTGATCCAGCGGAGGATCAGAAGACTGAGGACGACATTATGGACAGCTTCAACAAC

AGCAACAACCACCACCACCACAACAACATTCATTTCGCTTCTTCATCGCGTTCTAATCCCTCTGCTCATTTGAACACTTT

TTATTCCAACCAAATCACACCAAACATTGGACTATTGCAGCACCAAAACTCAGTTTTGATGCTGGACCAGTCCTTATCGC

GGATGTTAGTTGAAAACCAAGCACCAAACTTGAGGCGTAGTGCGAAAATAGAGTTCTCACCGGACACAGGGCTGAGCATT

GATGCTTCCTCAGTGGTTTCAAACAGGGAAATGGTACAGGACGATCCATCGTACTCGTCCGCTCCGATAGAACTCGATGG

CCTCTGGTATTATTGA

>MdNAC28 [Leaf=Malus domestica] NAC domain class transcription factor

TGGTTTTCGGTTCCGACTTGTACGAAATCCCGGTTCGCAAAAACAAAGGTTTATCCCACAAGTCGAGAGCTCAAATCTCA

AACTGTGACAGAAAACATCAACTGCATCATTGTCCATGTCTTCCTCCATGGCATCCTCAGCTTCCCAATCTGCTGATGAA

AACGAGGGGTTTGATCTGTTGAAGTGTTCGACCGTCCAAGAGGGCATGAAAGATGCGGAGATCAGGTTACCGTTAGGGTA

TCGATTTGATCCCACCGGAGATGAGATTCTTGTGTACTATCTGTTTAACAAGATTATGGACCGCGCGATGCCTACCTACG

ATCTTATAAAAGAGGTTGATGTGTACGAGTGTCATCCGAATCAGCTGCCGAATGGTGACTTCAGACATACTGCCGATTTC

AATGCTGCCTATTACTTCGCCAATAGAGAACCCTTCGACGCTCGTGAAGCCAAGATAATTAAGACGGCTACAGATGGTGG

TTACTGGAAGGTGATCGACGACGAAGAGGAGGTTTTGTTCAAGGACAGCGATGTAATCGTAGGGTTTGAAACCGTCATGA

AGTTCTACAAGGGGCAGGCGCCGAACGGAACCAAAACCCCCTTTGTCATGAACGAATACAGGCTCAATCCTCGTGTAGTA

CCTGCTCATGTGCTGAATGAAAGCATTAAGACTAAGATTGAGAGGTACGTGGTATGCCGAATTATAAACAAGGAGGTTTC

GAATCAACCAGCAATCGATTACGGACAGGGATTGCTCGAACTACTGCAAAAATCGTCTGCCGGCACCGTTGAAGATGGAG

TACCAAAGTGATGGGGCAGAAGCAGTCTAGTGCTTCATTGAAATAGCAAATGCAGCTCCTTCGTTTTGATGTTTTTCTTC

TAGTGTTATATAGATATAGCAGAACATTAAGTATCCAATATTTTTGGCCTAGTATTTGATTTGGCACAAGCGATTTTTAC

CTAGGAAATGGTAGGAGAGTAATTCAATTGATATGGGATTTGTTTTTTGGC

>MdNAC29 [Leaf=Malus domestica] NAC domain class transcription factor

ATGGGGAAAGGGAAATCATCGTTGCCTCCTGGTTTTCGGTTTTGTCCAACTGATGTAGAGCTTGTACAATATTATTTGAA

GAGGAAAGTAATGGGGAAAAGACTCCCTTATAACTTTGTTGCAGAGGTCGACATTCATAAGTATGCTCCTTGGGATCTTC

CAGACAAATCTAGTTGGCAAAGTGGAGATTTAAAATGGTACTTCTTTTGTCCGACAGAAAGGAAGTACCCAACTGGGGCT

AGAACGAAACGTACAACTGAATGTGGTTACTGGAAGGCCACAGGAAATGACAGATCTGTTCTTTACAATGGTGAAGTTGC

GGGCAAGATAAAAACATTGGTTTTTCATACAGGTCGAGCTCCAAAAGGAGAGCGAACAGACTGGGTTATGCATGAGTATA

GGCTTGAATCTAAGGACCTAGCTAATCGTGGTGTGCCTCAGGAATCGTATGTGCTCTGTACCATTTTTCAAAAAGAAGGG

CCAGGGCCAAGAAATGGTGCACAATATGGTGCGCCCCTTATGGAGGAAGACTGGAGTGATGATGAGGCTGAAAATTGTTC

AGAAGCAGTCGCACATGCAAATATGCCTGAACCAAACCTTGTGCTGCCGAGTAACTACAATAGTTCCATCACTACTAGCA

CGTATTCCCATGAAAGTATACACATACGTTCTTCATCTGAATCGTGCATATCAGATGCTGTACCACTTTCTTGCCATGTT

CCCCAACTGGTTTCCAGTAATCATGCTACAGTTGAGGAGCCTCATACTTCCAATGATGATGATATCCTGTCAATATTGGC

TTGCTTCTCGGAAGAAAGCCCTTCCTTAATCAAAGAAAACGAAAAAAATGAGGAGCTTGGTAATGTTGTTCCTATCGGAA

ATGCTAGTGCTACACCTCACGTCGTTAGTAATGATATTTACGTCACTTTAGGAGATTTGGGCAAGGTGGCTAGAGTAGGT

GAAGATGGATGTAGTTTCTCCAGTTTGCCTAATTCTGTCTGTGCTACGGGTCAAATGCCGCGAGGTGACGATGGGCAATA

TTTGGAGCTGGATGATCTTGGCGAACTATTTAATTACCATGATTCTACGCACACTCGGCCTCCTTCTATGTTCGGTGAGC

CTCAAACTTTGCTGGGAGAGATGCCTTTTCAGGGTGAGACCAGTTGAATGTGTTTGATAACGTGAACTTCTGAAATTGTT

ACTGCCCTCCAGTCTTTCAGATCAAAACTTGTTAACCGGTTTCGCCAACTTGCAGAAGTTCACCTTATCAAACACAGCAT

TCGAGTTAATTCTGCAAATTCTTTTTCATTGTTAAAACACAACAGTTGTAAATCCTAATTCAGTAGTTTTGTGTTGGAAC

TTGGATTTGGA

>MdNAC30 [Leaf=Malus domestica] NAC domain class transcription factor

ATGGGAGATAACCAGTTTAAGCTCCCTCCTGGTTTTCGATTTGACCCGACGGACGAAGAACTTGTAGTCCATTTCCTTCA

ACGTAAGGCAACTCTCTTACCTTTCCATCCTGATGTCATTCCCGATCTCCATCTCTACCCATATGATCCGTGGGAACTAA

ATGGTAAGGCTTTGAGCGAGGGCAAGCAGTGGTACTTCTACAGCAGGAAGGCACAGAATCGGGTCACGAGTAATGGGTAC

TGGAAAACATTAGGCATTGAAGAACCTATCTTTTCAAGCTCATCTTCTAATTCTACCAGCAAAGTTGGGATGAAAAGATA

TTTGACGTTTTATGTTGGAGAAGCTCCAAATTCAGGGATCAAAACTAATTGGATAATGCATGAGTATCGTCTCTCTTCGT

CAGCCTCAGATTGTGCTTCTTCTTCCACCACCACCACCACCACCACCAGATCTTCCAAAAGAAGAGGGCAACCCAAAACA

GAATACAGTGGATATATTTTGTGTCGAGTCTACGAGCGTGATGAAGAGGAGGATGATGATGATGATGGGACAGAGCTTTC

ATGCTTGGACGAAGTGTTCTTATCTTTGGACGATCTTGATGAAATAAGTTTGCCAAATTAATCATAATTAGAGTAATTAA

GAGAACTCATCAAGAGAGAAATTAGAGAAGAGAGAGGGACCTAACTAGTGCTCCATTATTACTGATGAGCTTTATCCAAT

TGTATAATTTCATTACTTCAAGTTGTAGCTAGATTAACGTTGTAAACAATCTCCATAAGTTACACTCCGAGTGGGTAGAC

TTATATGTCGTGTTGCAAACTGAAGCAAAAAAATTGTACGGAAGTTTAAAGTTT

>MdNAC31 [Leaf=Malus domestica] NAC domain class transcription factor

ACAATAAAAATTTAATCGAAAATACAAAACAGTTTCTTGTAATCTAAGGCATTCCCATCATTTCTTCACCACCAATCAAG

CCCCTACCTTTGCTTTTTCCCTCCACCAGAAGACTCCGTCCTATCAACCACACAATCTCTCCCCTTCTCTCAGCTTTTCG

GACTCATGGGTCGAGAAGCGGACCTTCAGATAATAGCATCAGCCGCCGCCGAAGTCGCCGTAGCCGCCCCCTTAGTGCCG

CCGACACTGCCACCGGCCCCGACTGCCCTCGCTCCCGGCTTCAGGTTCCACCCAACAGACGAAGAACTCGTCATTTACTA

CCTCAAGCGCAAGGTCTGTCGCAAACCCTTCAAATTTAACGCGATCTCTGAGGTCGACATCTACAAGAGCGAGCCCTGGG

ACCTCGCTGACAAGTCGAGCTTGAAGAGCAGGGACCAGGAGTACTACTTTTTCAGTGCATTGGATAGGAAGTATGGAAAT

GGGGCAAGGATGAATAGGGCTACCAACCAAGGGTACTGGAAGGCCACCGGAAATGACCGGCCCGTTAAGCACAACGATAT

CGTCGTGGGGATGAAGAAAACCTTGGTGTTCCATAGCGGCCGAGCTCCAGATGGGAAAAGGACCAATTGGGTTATGCATG

AGTACAGACTTGTTGATGAAGTTTTTGAGAAGGCTGGGCTCGGTGCCATTCAGGATGCGTTTGTGCTATGTCGAGTATTT

CACAAAAGCAATATAGGACCACCAAACGGGCATCGGTATGCGCCTTTTGTTGAGGAGGAGTGGGACGATGATGATAAGTT

AACTTTGGTTCCTGGACAAGAGACCCGGACTGTAGCTGTAGTTAGTCGTGATGCATTTGTGGTAGGAAATGGTCATGCTG

CATGTAGTGAACAAAATGATTATGCTGCTGGTAATGAACAAAAGGTACATGCTGCTGGTAGTGAACAAAAGGTACATGCT

GCTCGTAGTGAACAAAATGGACTTTCTGCATACATTAGAGGAAATGGACATGCTGCATATACTGGAGGAAATGGACATGC

TGCACATATTGGAGGAAATGGGCATGCTGTATATATTGGAGGAAATGGTCAAGCTGCATATAATGGAGAAAATGGTCATG

GAACTTCCGTTGAAGGAAATGGTCATGGAACTTCCATGGAAGGAATTGCTCATGGAACTTCCGTTGAAGGAACTGGTCAT

GGAACTTCTGTCAAAGGGAGTGGTCACAGCACTTCTGTCGAAGGACGTGGTTGTGACACTTCTGTTGAAAGAAATGGTCA

CGGCACTTCTGTCGAAGGAAGTGGTCATCGCACTTCTGTCGAAGAAAATGGTCATGGCATTTCTGTCGAAGGAAATGTTG

TTGAAGGAATTGGTCATGGCGCTATAATTGTAGTAGATGACAATGGAACTACTAATGAAGGTGACTGTCACGAAACTTGT

ACTGCAGGAAATGGTCACAGTGTGCCCAGTACAGAAAATAATATTGAGCAGAATACTCAGGCAATCAGCAAGGCTATTGT

TGTTGTTCCTGAACTTCCAGCGGAGAATCAAACTGTTTTACCACCATGCAAGACAGAGAAAACTGACGATTATCCTATGA

CATGTGTGGTTAATAGAGAAGAGAGATTGGATGACTATCCGTCACCAGGTCCAGATGATGCGCAACCCCTGCTAACTTTG

TTTAATCGACAACCTGGGCAACTACGGCAGTATAAAAGAAGGCGGCATAATGAGTCAAATTCTAACCATTCAAATGCTTC

TGAGATTTCAAGTGGGATGACACATGACCCTTGCTCATCTACAACAACAACAGCGTCAACTGAGGCATCGATGACGACTA

CCAGAAATTTTCTCTCTGCACTGGTGGAGTATCAGCTGTTAGAATCCCTTGAACCCAAAGACACTACCCCAGCACCTCCT

CCTGAACTCAATGCTGCTTTGATGGAATCTTCTGTGCCTACCAGCTGCTTGAAATACATAGAGACTTTACAAACTGAGAT

CCACAAAATCTCAATTGAGAGGGAGACATTGAAGTTTGAAATGATGAGTGCCCAAGCCATGATCAACATTCTTCAAGCCC

GAATTGATCTCCTAAACAAGGAGAACGAGGATTTGAAGAAGAAGGTCTGAGTCGCCTAGTGGATTCGTAGTGGTATGTAC

TCCATAGTGTTCGTTGTATGAGTTATCTGCCCCTTATGGGCCTCGTTGAACTATCTACAGTGAGTATGTTTTCTCTATTA

GAGTAAGAGCTGTCTCTGTTGTTAATCGAACGATGTTGTAACCGTGTAGGAAGTTTGCTGTACCGGTAGCAAGCTGAACT

GTGTTGTACCTGTCATCGAAATCGGCAGTTTTAGACCCTAATTATTATAATGTTCGAGCTTATTGCCTTTGTTAGTTAAC

CAAAGTAATTGCTTAGACAGTGGTTACTTGAATGCTGGGACTGCAATTCCCAG

>MdNAC32 [Leaf=Malus domestica] NAC domain class transcription factor

ATGGCTCCTGTTTCATTGCCTCCTGGTTTTAGGTTCCATCCCACAGATGAGGAATTAGTTGCTTACTACCTCAAAAGAAA

GATTAATGGCCGTAAGATCGAATTAGAGGTCATCCCTGAAGTTGATCTCTACAAGTGTGAGCCATGGGACTTACCAGGAA

AGTCATTATTGCCTAGCAAAGATCTGGAGTGGTATTTCTTCAGTCCGCGAGATCGGAAGTACCCTAACGGATCGAGGACT

AATCGTGCAACTAAAGCTGGATACTGGAAGGCGACGGGAAAGGATCGGAAGGTAAACTCACAGACGCGTGCTGTAGGCAT

GAAGAAAACCCTAGTTTATTACGGAGGAAGGGCACCACATGGGAGTCGAACAGATTGGGTTATGCATGAATATCGCCTCG

ACGAGAGGGAGTGCGAAACTGCACAAGGCTTGCAGGTTCAGGACGCATATGCGCTATGCCGTATTTTTAAGAAGACTGCA

ACGGGGCCAAAGATAGGAGAGCATTATGGTAGCACAAGTACTACTAATTACCAGCTAACCAGTGACCATTCATCTAGCGT

TAATGAACTGTATTCTGATGGAGGAAGATGTGAAGATTTCGAGAGCTCAAGTTATCCTATGCAAATGAACAATGCATGCT

CTTCATCACCAAACTTTGTTCATACAAACTCACTTGATGACATGGGTAGAAAAAGAGATGGAAAATGGACGCAGTTCTTA

TCGAAAGAGGCATTTAATTGCTCGTCCTCATTTTCTCCATTTCCCAATCATGAAAACATTTCATACCCTCCATCTAAGGT

TGACATAGCATTAGAGTGTGCAAGGCTGCAGCATCGACTCTCACTTCCTCCATTAGAAGTGGAGGATTTCCCTCAAGTTG

GACTCAACGACTTCAAAACGATGCAATACTCAAATCCTGCACTTGACACCACAAGTACTGAAACTGATGCTTTGCAGGAA

ATTCTTTCAGTTGCACATGTTTCTCAAGAAATGGTCAATCAATCCAGTAATCATGTGGATCAGACATGGGGTGGAAACTA

TGCACCTCCAGCCAATGACTTTAGCTTTATGGTTGATCGAGATGCACATTATAATCAGATTACTGATCACTTGAATTCTA

TGAGATATGTGGATCAAAAAACATGGGGAAATTCGTATACACGGTCAATCGAGATTGGAGACCTGGAGGATGGTTTTAGG

ATGGAGAATACAGCCGAGAATCTAAGATGGATAGGAATGTCAGACAAAGATCTGGAGAAGAGTTTCACGGAGGAACACAA

GATAGTTCCAATAGAAAACATTTCATCTTTTCGTAGGGAAGAACAAGAGCAACAGTATGAGATTCAAGGAGAAACTGGGC

ATCATAGTGGCATAATCAAGGAACTCAATGATAATGAGGCAAACGATGATGATTTCTCACTTAGTTTCATCAATGAAGAC

CCGAACGAGAACTTCCTCCATGATGGAAACATGGATGATTATTCGCCTTCTCCAAGCTTTGAAGTCATCGAAGAAATTCA

AGTTAATCACGGCATGTTTGTTTCAACTCGGCAGGTGGCTGAAACATTCTTTCACCAGTTAATGCCTTCACAAACAGTTA

AGGTCCATCTAAACCCAGTGTTCGGCCAGAACCTTTTGGTAGAGAGAGTTGTTACACAAACAAAGTCCAAGGATAGAGGT

TCTTTCTTTGAGAGGTTCAAGGAACTTGTGATGGAAAAGTTTGACGGAATTGCCAAATCAACAAAGCCATGGAGGAAAAT

TGCAAGCACTCTTGTTTGTGCAGTTACACTTATATTGATGCACATGATCATCTATCTTGGGCAACATATGGAAGATGAGA

AATTCATGGATGCCTTCACCACAACTACAACTGCAACGATTGTGGAAGAAAAAGGCAATTCTAACATTAGCAATAAGAAG

GAAGGACTTGGATTAATTAAGTGGAGCAACAAAAAAGAAAAGGTTTGCTTGGTTAATATAAGAGATGGGAGTACTTGTAG

TGTGTTTCTGAAGAAGATGGGGATTTTCCTCACAGTTTCTTTGGCTCTTGGTACCATGTGGGCTAACCACAATGTAATTC

CCTCTTGAACTCAACCATTTTATTTCGGTTCTAACAATAGTTTGTCTGTTTGGCAGAGGGGAAAATTCAGCCTAACCCAT

AGCTATAAGTTCAAATATG

>MdNAC33 [Leaf=Malus domestica] NAC domain class transcription factor

CACACACGCACACACGCACACATACGCTGAGAGAAAAGAAACACATGGAAACACCGGGAAGAAAGAGAAAAACAAGAGGA

GGAGGGGGTTCAGGGCAGGTAGGGTTCAGATTCCACCCCACCGGGGAGGAACTGGTGGACCATTACTTGAAGCTCAAGAA

ACAGGACAAGGATTTCCAAGCTGAAAACATCCCTGAAGTCGATGTCTGCAACTTCGACCCTGGGGATTTGGCTGCTCGCA

TGCCATCCGACGATATGGAGTGGTACTTCTTCGGTCGAAAGAATTATAAGTACAAGAACAGCAAGCGGTCCAACAGAACC

ACACCAGGAGGCTACTGGAAAATCACAGGCAAGGAGCGTGATATCAAGGCTCGGCGGTCCAAAGCTGTCATTGGTAAGAA

GAGGACCTTGACGTTCTACCGGCGTTGTGAGCCTAAACCGAAAAAAACCAACTGGGTCATGCACGAGCACGATCGCATTG

ATAGTGAAGCCAATCCTAAACTGCCTCAGGATTTCGTTATCTGTCGCATGAAGAAAAAATCCGATGAGGAGGATACCTCA

ATCGGTGAAGTTGAACTTGGCAGCTGTAGTGTGTCTAATGTTGAAGATCATGCTGCAGCTGTTGTGACTCCAGGGTCACA

GGATAACAGTTCCTCTACACCCCTATCATCCGGATACATAGAGCTGGAAGATGTTCTGCAAGCCAATGGCACCAATGATG

ATTGTAATGCAATACAATCACCATTTGGAGATAATAATTATTCTTATATTAATAAGAATGATATTTCAACCTGTGATGAA

GGTGAACCTCATAGTTTCACCGTATCTGATTTAAAAAATGAAGCTCCACATAATATGTCTCAACAGTCCCAAAACGACAT

GGATTCACTCTGTTTACATGTCGCCCAAGAGTCACCAACATGTGTTATATCCAAGAATAATGTTTCTACCAGTGATGATG

ATGAACAATTTAAGAACTCCATTTCTAATTTTGAAAATCAAGCTACGAATAAAAGGATTTCAGAGGTATGTCCTCCACCA

GAAGAAGATATGGAATTTTTCTTTGATCTACCATTTTTCTTTGATCTACCTCAACTAGAGGACTACACACTGTAGCCACC

AATGAAAACAACAGTGGGAGATGTTTTGCATGACACAAGCTATCGAAGTGCAGGAGTGGTACGAACACCAAAGTAGTGTC

ATGGATGTGTAAAATGCTATTATGCATTAGCTAAAGGAGAATAGTAAAGAGGGCTTTTGAATTAGGCATGGAAAAGGTAG

CTCACAGTAACTGAAGGCCTTCTTTCAAGTTCGTGGAAATATCTCCCTGAAGTCGTTAATCAAATCCTCCACGGAATAAT

GTTTCGACCAAGCCATAATG

>MdNAC34 [Leaf=Malus domestica] NAC domain class transcription factor

CGAAATCTGAGAAGAAGCAAATTAGCAAGAGCAAGAGGAAAGAGAAGAAGAAAGGAAAAGGGAAGGAGTAAAAGCGAAAC

AGTAAAAAGTAATCATGAGCGGAGGAGGAGACGAGCAGCAGCAGCAGCAGCTGGAGTTACCGCCGGGGTTCAGATTTCAC

CCGACGGATGAGGAACTGGTGAACCATTACTTGTGCAGGAAATGCGCTTCACAGCCTCTCGCTGTTCCAATCATCAGAGA

AATTGATCTTTACAAATTCGATCCGTGGCAGCTCCCTGAAATGGCACTTTACGGAGAGAAAGAATGGTATTTCTTTTCGC

CAAGGGACAGAAAATATCCAAACGGTTCAAGGCCGAACCGGGCAGCCGGAACCGGGTACTGGAAGGCGACGGGGGCTGAC

AAGCACATTGGAAAACCCAAGGCACTCGGGATTAAAAAGGCACTCGTGTTCTACGCTGGTAAAGCCCCCAAAGGAATCAA

AACCAATTGGATCATGCACGAGTACCGCCTCGCCAATGTCGACCGGTCCGCCGCCGCTGCCAAGAAAAATCAAAACCTGA

GGCTTGATGATTGGGTACTATGCCGCATATACAACAAGAAGGGCAGCATAGAGAAATACAATGTTACAACCAAAATGACC

AAATACCCAGAAATATCGGACGAGCAAAAACCTGACATGACTATAATGCCCCCGCCGCCGCATGCAGCATCAAACACGCA

TCACATGATGGATTCGTCGATGGATTCAGTACCGAGGTTGCCACAGACGACGACGGACTACTCGAGCTGCTCGGAGCACG

TGCTGTCACCGGAGGTCACGTGGGAGAAGGAGGTCCAAAGTGAACTGAAATGGAGCAATCAATTGGAGAATTCCTTCAAT

ACCCTCGACAATCAGTTCCTGAATTACATGGATGGCTTCTCGGATATTATGGACCCTTTTGGGGGGCAATTGCAAATGGA

GCAGCAGCAGCCCTACCAGTTGCAGGACATGTACTCCTATCTCCACACCCAATTTTAAGCCAACTTACAAGGGTACAATT

GGAAAATGGCAAGACGGAGAGAACAAGAAAAAAAAAAAAAAGAGGACCATGATTATTTGATTTAGTACTAGACTACTAGT

TAATATTCTTTTGAATTTGGTTACATGATCAATTTAATTTTATTTTGGATGTCTGCAAAAAAAAAAAAGGGGGACGTTTA

GATAATGTTAATATGTAATGAGATTCCTGTAGAAAATTTTCATAATTTGTTCCAAGTTATGTACCATAGAAAATTGTTTT

ATTTTTCTTTTAGAACGTAAGATATGAATGAGAAATTTTTTCTCGAAAAAAAA

>MdNAC35 [Leaf=Malus domestica] NAC domain class transcription factor

CAATCAGTAACAACAAAACTAACTAGCTTGCGCCGTGCTTTGATATCAATTATATGGCGCCAGTGGGATTACCTCCCGGT

TTTAGGTTCCATCCAACGGACGAGGAGCTGGTGAATTATTATCTCAAGAGGAAAATCAACGGCCAAGAGATTGAGCTCGA

CATCATTCCTGAAGTTGATCTCTACAAATGCGAGCCTTGGGAATTAGCAGAAAAATCGTTTTTGCCAAGTAGAGACCCCG

AGTGGTACTTCTTTGGACCCCGGGATCGAAAATACCCTAATGGATTCAGAACAAACAGAGCAACGAGAGCAGGATATTGG

AAGTCGACTGGGAAAGACAGGAGAGTGACGTGCCAGAATCGAGCAATCGGAATGAAGAAGACGCTGGTTTACTACAGAGG

CCGAGCCCCTCAGGGCATTCGGACTGACTGGGTCATGCACGAGTATCGCCTCGATGACAAGGACTACTACTGCTCCTCCG

ACCTCGACACCTCCTCCTCATCATCTGCCGCAATTCAGGATTCTTTTGCATTGTGTCGCGTGTTTAAGAAAAATGGCATT

TGCGCGGAGATTGAAGAGCAGCAAGGCCAATCAAGTCATTTAACACTAAACAATATTCATGAAAGATCACAGGGTGTAAA

CAATGATCACTGTGATCAAACCCTGTCACCGGAGAATCCGATGGCATCGTCATCGTCAATCTCATGCCCGCAGGAGGAAG

ATGACAAAGACGATTCGTGGATGCAGTTCATCACAGATGATACGTGGATGCAGTGTTCTTCTAACGCTGGTGTCAGTGGT

GAAGATCAGCTCTCTCATGTGGCCTTCACAAACTAACTGATCAATGGAGATGAGTATATGGATGGTTACCGGAATATTGA

TGTTGGCGGTAAATAACCTCTTGAATGGCATGTTAATCTTCACAGTTTCCACTGCTTGAATTCTGCAGAGCCTAACATGA

AGGAACTAGCATTTCGATGCCCACCGCCTCCAAATTCCTGTGAGATGAGTGTTGTGTCTT

>MdNAC36 [Leaf=Malus domestica] NAC domain class transcription factor

ATGGCGCCTGAAAACATGAGTATATCTGTAAATGGGCAATCTCAAGTCCCTCCTGGATTCAGATTTCATCCAACCGAAGA

GGAACTCTTGCAGTACTACTTGAAGAAGAAGGTTTCAAATCAGAGGATTGATCTCGATGTCATTCGTGATGTCGATCTCA

ATAAGCTTGAGCCATGGGATATACAAGAGAAATGTAAGATAGGAACTACACCGCAGAATGATTGGTATTTCTTCAGCCAT

AAGGACAAGAAGTACCCGACTGGAACACGAACAAATCGTGCAACTGCTGCCGGATTCTGGAAGGCAACCGGCCGTGATAA

AGTGATATGCAGCAACTGCAGGCGTATCGGTATGCGGAAGACTCTGGTGTTCTACAAAGGCCGAGCTCCCCATGGCCAAA

AGTCTGATTGGATCATGCATGAATATAGACTCGACGACAATAATACTAGCAATTGCAATATCACTAATGTGTCCACAGTT

ATGGGAGAGGCAGCACAAGATGAGGGATGGGTGGTTTGCCGAATCTTCAAGAAGAAAAACCTCCACAAAAGTTTGAGCAG

CCCAATCTTGAGTACTACTACTTCATCCATCACAACAGAAACAAGAAGTGGCCAATCACTGTTTGATTCGTGCCCCGAGG

GAACTTTGGAGCAAATACTTCAATACATGGAAAGGACATGCAAGGAAGAAGAGAATGAAGCCTACATTAACAATATTAGC

ACAAGATTTAACCTCCAACCAATCAACACTGGCATTAGTAATACTACCAATCACAATGGCTTCCATGAGAGGTTCTTGAA

ACTTCCAACCCTAGACAGCCCAAACTCCACAAGCAGCCAGGATTGTTACCAACCAAACATTCATGAGGAGATGATGGTCA

CAGAGAAGAATAATGACCAGGTGAGTCCCTTCACTGATAATCATCAAAACATGGATTATAATGCACACCACATGGACTCA

GGACTCACCAGCTGGGAAGCTCTCGACCACCTTGTAGCTTCACAGCTCAATGGACAAACCGAAGCCTCTAGGCAATTAGC

TTGTTTCAGCTCTGACCCCCACAACACTATTGTTTATGACAACGATGATCATGATCATGAACTCCAATTACCAAGTACCC

TACGAGGATCATTGTCTTCATCCAATAAATCCTACCATCCCACTCACCATTACAACAACAGCGAATTCGACCTGTGGAAT

TTCGATCGACGATCAGCATCGTTGTCATCCTCCGACACGCTGTGCCACGTGTCGACCGGTCCTATATAA

>MdNAC37 [Leaf=Malus domestica] NAC domain class transcription factor

ATGGCTTCCAATGGAATCCGCTTCACCAGAGTAGAAGTAGGAGGCATGTTACTGCAAGTGGGATACCGATTCCACCCTAC

AAAAGAAGAGCTGATCAGCCATTACTTGAAGTTGAAGCTCCGGGGAATGGATTCCCTCGTCAGCGACGCCATCCGTGAAA

TCAATATCTGCAACTACGAGCCCTGGGAATTGCCTGAGATTTCACTGATAAAGTCGGATGATGAATGGTACTTCTACAAC

CGACCAATTTACAAAAAAAACAGCAGAAACGAAGTTGAAAGGGCGACGGCCACAGGCTTCTGGAAGAGCACTGGGAAGGA

AAAAATAGTCAAGGCTCGAGACAACAAAATTATCGGTAAAAAGAGGATTCTGACATTTCGCCAAGGTCGCGGTCGTAACG

CCCCAATTACCAACTGGGTCATGCATGAGTTTTCCATCCCTCAAATCAATCCTAATGCTAATCAGAGGGATTTCGTTCTC

TGTTACGTAAAGAAGAAATCAGGTAAGAACACGGATGTTGCAACCGGTGATGAAGGTGAATCTAGTAATTACAACAATGC

ACATGACTTTGAAAATCAACTGCAACCAGCACATAATATGCATGTTGAAGAGGAACGTACTCAGCCACCACCAAATCCGG

ACATTTTTCAGAGGGAATTGGATAGTATGCTTGGACATACGCGTGGTAATAATGATTACCATGGAATGGATGAAAATTTG

GAAGAGTTCGTAAATGGACTTTTTGGGGATGACCCGTATCAGGGTTACAGTGAAGAAATAACTCACAATAACGTCTTTAA

CGACTCCACTCTGCCACAGTCACCTAGGAAGGTGTATTTACCGGATTGTGGAGTGAGCAGTGACTCAGACACAGAAGTAC

AACATCAAGCTGCACAGGGCCCGAGTTTACAAATGCTTGGTGTGCCACAAGCTAGTTCGAGCCACTTCGGAAGAGAGGAA

GGTGCTATGCTTCGTAACCAGACCTTAAGACAGGCTGCTTCCTCTATCAATGTGTTACCTAAACCTCAGACTGATCGTCT

TCAATCAATCAGTGATGAAGATTCTGGTACACACCAAATAACAAGCAGGCTGCGACGTGCATCAAGACCCATTAATGCCT

TAAAATTAAAGAGTGCTTCCTCTGTGGATGTAGATGCAGTTAGCCTTCAGATTAACTCTATTCAGTTAGCCTCCGACGAA

TACTATAACAGTGAGAGAACACGCAGAAGAACATATCCAACAAGTGCCCTCTTAGCACTAGGCAAACCTAATGAATTAGA

ACAGCAGCAAAGCAAGGCCAAAGTAGCTGCAGAGCGTCGAGCTGCTGTTGATTTTCCCCCGAAGAGGAACTTTATAACAG

AATCTAACAAAGACGCAGAGGCGGTTCAAGGCAATAATACAGAAAAGGGTTTGAAGCAGACACACAATGCAACCACCGCC

GGCAATTGGATGAGCTGTTCTTTCATTTCCTGGGAAACTTCCCCCCCATTAACAAGTCCCCCATCAGTATACCTTTTCAA

CACGGTTCTAGGTGCGATATTGTTCTTTGTTTTTGCTTGGGAAGAAGGTTTATACGGGCAGTGGTGCTAGATGAGCGTCA

CACAATGAGTAATGCCAGACTTAGTGGTTGCTTATTGCTGCTATAAAATGTGGGAGTATCAGCCATGTTTTGCAGTGATG

AAATGTTTACATTTGGCATAGCCTATGGAGTTTGACATGTTTCTGTTTCTACGGACCAGAAGTAATGATGTATGGCTGGT

ACTGGAATCTCAGTACGGTATTGTTTTTTTGTTTAATCATTTGGGAAGCAGAAATTCGAACTCTTGGGATATTTTCTTAA

AATTGTAAAAGATAAATACT

>MdNAC38 [Leaf=Malus domestica] NAC domain class transcription factor

ATGGAGGTGGGTTATTACGGTATGCCATTGGGTTTTCGGTTTCATCCTACTGATCAAGAATTGGTCAGTTCCTATCTTCG

CGATAGAGCCCTCTCCGGAAAGCCCTTAGGCAACCCGTTTGTTCACGAGTTCAATCTCTTTGGACAAACACCACCCTGGG

TGGTTTGGGAGCAATTCGGTGGAAATTCCCTACTCGATCAGGACTTGTTCTTCTTCTATGAGCTGCGTTCTAGAAGTGAC

AGCGACAGCCGCAGCAAACGCCAGATCGAAGCCGGAGGCACTTGGAGCGAAACATCCTCCGACAAAGTTTTAGGTTTGGA

CGGAATTCCAATCGCGGATAAAGGGCATTTCAGGTATAACAACAAGGGTTCTAACAACAATGGCGGCTGGCTGTTAGAAG

AGTACAGTCTTCTTCCTAATGTAGTTGCTGGCGCAAAGGTGGTTCTTTCTCGACTTAAAAGGAATCCGAGGTCTAGGTGC

GGAAACAAGATGAATTCCTCAAGTCCAACTCATGACTTCAATGAGAAGCCACCGATTAAGAGGGCAAGAAAAGAAGTATC

GAATGAGAAGCCGCTGATTAAGAGAAAGAGAGCAAGAAAAGAACCGGGTTCACAAAAACATTCTAATGTGAATGTGATCA

CCAATACCCCTGCTGTTTTGCCTTTTGTTGGAACTGAAAGCAATATGATTACTGACACTAGGTATCAGGATCAGGAGTGC

ATAACCAACATTAACGGCAATACGAAGCCGACTGATTTCTCTGATCAGGATAGTGTGATATTGATGAGTGATGTTCCTTC

TTATCCCATATGCTTGGACGGCGACACTAGCAATCACGACCAGGGGTGCATAAGCTACATTAACGGAAATGTGATGCCCA

TTCCCACTCATATTTCAGATCATGAATATGTGCCACTGATGAGTGATGTTCCTTCTTCTCCAATATTCTTGGGCGGCGGG

GAATATCCTTTAGACGGTGAAGCAGTGCGTGGAGACATTAAAGAATGGTTGACAACGCTTGAAGGTAAATTGCCCCGGGA

GTGGCCTCAAATTGACGAATTCAGCCAACCGTTAGACGGGTTCAATTACTGGACAGACTGTTTGTGGATGAACGATTCTG

TGAATGATCAATAG

>MdNAC39 [Leaf=Malus domestica] NAC domain class transcription factor

TTAATATCTTCTTCCATTAAGCAAAAACCACCCGCTGAAAGCCGAACACTTTGATTAAACCCCAAGTCATTGTCCGTACT

GTTTCTCCTGTGTTTCTAATCCTATACAAATCCACCCAGATTCCGATTCTCCTTCATGAAAGATAGATGGCTGCCAATAG

AAACCGCTTCACCAGAGCAGCAGCAGGTGACCTGTCGATGCCAGTGGGATTCCGGTTCCGCCCTACAGAAGAAGAGCTGA

TCGACCATTACTTGCTGATGAAGCTCGAGGGTAAGGATTACCTTGTCAGCGACGTCATCCGTGAAATCAATATCTGCAAC

CACGAGCCAAGGGACTTGCCTGGGTTTTCACTCTTAAAGTCGGATGATCAAGAATGGTACTTCTTCAGCACTTACAAAAA

AAACAGCAGAAACCAGACCGAAAGGGCAACGAAGGAAGGCTTCTGGAAGATCACTGGTGCCCAAAAACCGGTCAAGACTC

AAGACAATAGAACTATCATCGGTAAAAAAAGGATTCTAACTTTTTACCGAGGTACTGTTCGTAATTCCCAAAGGACCAAC

TGGGGTATGCATGAGTATTACATCCCCCAAACCAATCCTAATGCTAATCAGAGGGATTTCGTTCTCTGTTGCGTAAAGAA

GAAATTGGGTGAGAATGTGGATGCTGCAAACTGGGATGTTGAATCTATTACTTACAACAATGAATCTGATTTCGAAAATC

AACTGCTACGAGCACTTGGTATGGATATTGAAGAGGAGCATACTCAGCAACAACAAAGCATGGACTATTTTGAGAGGGAA

GAGGGTCGTATGCTTGCAAGTGCCCCTGGTAATAATGATTACCATGGGTTGCAATCTGCATTTGGAGCTGATGAACCGGA

TGATAAGTTCGCAGAGTTCTTAAATTCAATTATTGTTGATGGGGATCAGGATGAAACAACTCATGCTAACATCTTCAATG

ACTCCACTCATCCTGATACAACTCATGCTAACATCTTCAACGACTCCACTCTGCCACAGTCAATTATGAGCGTGTATTTT

GAGGATGAAGCATTAAGCAGTGACGCAGACACAGAAGTACTCCTTGCACAGGAAATGAGTTTACAAATGCTTAGTGAGCC

ACCAGTTGATTCGAGCCACTCCAGAAGACAGGAAGGTGTTATGCTTTACCAGACACAGGCTGCTTCCTCTGTCAATGTAG

TACCTAAACCTCAGACTGATCATCTTCAAGTAGTCACTGATGAACATTCTGTTACACATCGTGGTCAATTAGTTAATGAC

GAGCATTCTGGTACACATCAAAGAACAAGCAGGCCGCAACGTGAATCAAGACACAATAATGCCTTAAACGTAAAGAATGC

TTCCTCTGTGGATGTAGATGCAGAATTACGTCAGATTAAATGTATTCGATTAGCCCCTGACGAATACTATAACAATGAAA

GAACACGTGGAAGAACATATCCAACAAGTGCCCTCGAAGCACTAAGCAAACAGAAAGAATTAAAACAGCAGCAAAGCAAG

GCAAAAGAAGCTGCAGAGCTTCGAGCTGCTGTTGATTTTCCTCCGAAGTTGACATCTATAACAAAGTCTAACAAAGAAGC

AGAGGTGGCTCAAGGTAATAATGCAGAAAAGGGTGTGAAGCAGACACAGAACACAACAACCACTTGCAATTGGAAGGGCT

GTTCTTTCATTTCATGGGAGACATCCCCCCCATTAACAAGTCCCCCATCAGAATACTTTTTCAACATGGTTCTAGGCATA

ATAATGTTCTGCTTTTTTGCTTGGCTAGTAGTTTTATACGGGCAGTGGTGCTAGAGGGGTTCATTCACCAGAAAAATGGT

TAGTTTTTGTTGTCTGTCACACGATGAGTAATGCCATACTTACTGGTTACTTATTGCTGCTCTAAAATGTGGGAGTACCA

GCCACGGTTTGCAGTGATGAAGTGTTTATATTTGGTATAGCCTATAGAGTTTGACTTTTTTCTGTTTCTACGGACCAGAT

GTAATGATGGATGGCTAGTCCACATAATATATTGTTTTTTGCTTAATCGTTTTGGGAGCAGGAATTCGAACTTTTGGGGA

TATCTTCATAAAATTGTGAAATAGAAGCACTACTAG

>MdNAC54 [Leaf=Malus domestica] NAC domain class transcription factor

CTCTCTCTCTCTCTCTCAAATTTTATTTATTTTCTGCTGTTGTTCTACGGAGTTCTACTACTACTTCTTCTACTTGAATT

TGTCTTCTTCTTCTTCATTGCTTTCTGTCTTTTAACGAGTAATTACACCAACAACAAAAGTTGATCAGTAGTAAGCATTA

CAAACTACCAGCTGCGCTATGATGGAGTCAATCGAGTCTACTGTCCCACCCGGTTTCCGCTTTCATCCGACGGATGAAGA

GCTCGTCGGGTACTATCTTAGAAAGAAAGTTGCCTCCCAGAAGATCGATCTTGATGTCATCAGAGACATTGATCTCTACA

GAATCGAACCATGGGATCTTCAAGAGAGATGTCGGATCGGGTATGAAGAGCAGAACGAGTGGTACTTCTTTAGCCACAAA

GACAAGAAGTATCCAACTGGGACAAGGACCAACAGAGCAACCATGGCTGGGTTTTGGAAAGCAACGGGTCGAGACAAATC

AGTATACGATAAGGCAAAACTGATCGGGATGAGGAAAACTCTTGTCTTCTACAAAGGAAGGGCTCCAAATGGACAGAAGA

CTGATTGGATCATGCACGAATACAGACTCGAATCTGACGAAAATGGACCTCCGCAGGAAGAAGGATGGGTGGTATGCAGA

GCATTCAAGAAGAGAATCAGTAGCCAAAACAAAAGCATTGAAGGCTGGGACTCAAGCTACTTCTACGAAGAACTTAACGG

TCTCACTTCCGTCGCTGATCCAACGGATTTTATGATCTCGAGGCAGCCCCAGAGCTTTTTAAGCCAGAATTTCATGTGCA

AGCAAGAGACAGAAGCTGACAGTTTGAACTTCCTGCACTCTGATCCGTTTGTCCAGCTTCCTCAGCTTGAGAGCCCGTCT

CTTCCCTTAATAAAGCGGCCGACCTCAATGTCCCTTATCTCTGAGAACAATACTGAGTTAGAAGACGAGCAAAGTATTCG

AGGGTGTAACAATGCGGAGAAGGTTACTGACTGGAGGGCTCTGGACAAGTTTGTGGCTTCACAGCTGAGTCAAGAGGAGA

GGTATGAAGGTGATGGAGAGGCAAGCTTTGGTGCACATGATGAATCAGATATGTCATTGTTGTTGTTGCAGAGTAGTAGT

GCTAGAGATGATGATGATCAAGTGGAGAACAAGTTAAATGGGTTCCTGAATTCGAGTCCTGACTGTGATATTGGGATATG

CATATTTGAAAAATAATGAAGCAAGAGGGACAACACTTGGGGGAAGGTAATTTTATTGTTACGCACGTAGGTATTATAAA

TGGTATAAGGGTTGATGAATGTCGATCCTAAGACATATTTTTGTAAAAATATGCTTGTGAGTGGATTTCTCTCTCTTTTG

CACACCCCTAACACACATTCACA

>MdNAC55 [Leaf=Malus domestica] NAC domain class transcription factor

TTCGATTATCTGCTCTCCATCTTCAGTTCGCTCATCACCACCAATATTTT

GTCTCTTTGATTTCTTTTTTCTTGTGTATAATGTCTGATGATCATATGAG

TCTATCAATAAATGGTCAATCTCAAGTTCCTCCAGGTTTCCGATTCCATC

CAACCGAAGAGGAGCTTCTTCACTACTATCTTAGGAAGAAAGTTGCCTTT

GAGAGGATTGATCTTGATGTAATTCGAGAAGTTGATCTTAATAAGCTTGA

GCCATGGGACATTCAAGAGAAGTGCAAAATAGGTTCCACTCCACAAAATG

ATTGGTACTTCTTCAGTCACAAGGACAAGAAATACCCAACCGGAACTCGA

ACCAATCGGGCAACGACTGCCGGGTTTTGGAAGGCCACCGGGCGGGACAA

GATCATCTATAGCGGCTTCAAAAGAATTGGATTGAGAAAGACACTTGTGT

TTTATAAGGGTCGAGCTCCTCATGGACAAAAGTCAGATTGGATCATGCAT

GAATATAGGCTTGAGGAAAGCAACAGCACTCATGACACCACCGTTTCTAG

CTCAATGGGGGAGTCGATGACCGAAGAGGGGTGGGTGGTTTGCCGAGTAT

TCAAGAAGAAGAACTATCAGAAAGCCTTAGAGAGCCCTAAAGCCTCATTC

TCCATGGACTCATCAAACAACCAAATGCATGGTTCAAGAAACGATGGTGT

TCTTGATCAAATACTAATGTACATGGGAAGAACTTGCAAGCTCGAAAATC

ATGATGAACCCTTAACCATGAATAACATCTCAGAAAGATTTATGCATCTG

CCAAGGCTTGAGAGCCCAACTCTTCCAAACCTTCCCGCTTTCGATCAGGA

TCGTAGCTTCAAAGCTTGCTATCAGGCCATTGATGACATGTTCATAGAAA

CTGAGCCGCCTTCAACAAACCAACAAAGCAATGGTTGCGACAATAATGAG

CTAGTTGATGATCACGAGGACCCCAAAAGAAGGGTAAATGACTGGGTTAC

CCTTGATAGGCTTGTGGCATCCCAACTAGGTCAACTCAATGGCCAAGATC

AAGTGACACCAAAGCACTTGTCTTGCTTTGGAGATCCAAACATGGCCTTT

TGTTCTTCCCCTCCTCCTCGTAATGATCATGACCATGACGTACAACTATC

ATATCCATACCTACGTACAAGTAGTTCATCCCATCATCAATCCGACGTAT

ACAACAACGAGAATGATCTGTGGAACTTCACCAAATCGTCGTCATCACCG

TCATCAACGGACCCGCTTTGCCACTTGTCGGTGTAAGGGATGCACTTGAA

ACGACATATATACTATGTACGATATAGGAGCTTAACCTATAAGAAATATA

TATAGCGAGGAAGTACGTACGTATATGTTTACATGGG

>MdNAC56 [Leaf=Malus domestica] NAC domain class transcription factor

CAGAGTCCAATAAGTTCCAAGAAATAAAGGCCTTTGCATTTTTCATTTTT

CAAAATAATGGACAAGTTCAAATTTGTTAGAAATGGGATGATCAGATTGC

CTCCTGGTTTCCGATTCCAACCAACAGATGAAGAGCTAGTTTTTCAGTAC

CTGAGATGCAAAGTCTTCTCATGCCCACTTCCTGCTTCCATTATTCCCGA

GATCAACGTTTGCATGTATGATCCTTGGGATTTGCCAGGTAATTTGGAAC

AAGAGAGGTATTTCTTCAGCAACAAGGAGTCAAAATACCGGAATGGAAAC

CGAGCCAACAGGGTGACAAGTTCCGGTTACTGGAAAGCAACCGGCGCAGA

TAAAAAGATTGTATCTTCAAGGAGGAATCATATTGTGGGAAAGAAAAAGA

CTCTAGTATTTTACAGAGGGAAGTCTCCACATGTTTCTAAGACTGATTGG

GTCATGCATGAATATTGCCTTGTGAATGCAGAAACTACAGCTTCCATTCA

CGCAACTGAGAATGCTTTAACGCCAAAAGGAAATTGGGTTTTGTGTCGGG

TTTTTTCTAAGAAAAGAAGTGGCAAGATGAATGAGGAGATTGTGGTGAAT

TACAACAGCATCAAAGTTAACAATAATGCGAATCCTGCATCTTCTTCCTC

CTCTTGTTCAAGTTCAAGTGGGATCACAGAAGTAACTTCCCCAAGTGAAG

AATGTGGTGAAGAAATCAGTAGCTGCCCTAAATTTTGATCATATTAATTA

AATGTAGCATCGTTCTTCGTGTGTCGTGTTCGATCATGATAGGAAATGAA

TCTTTTTTTCTTGGAAGATATTAAGAGTATTCATACTATGTTTTTGTACC

ACATTTTCATACC

>MdNAC57 [Leaf=Malus domestica] NAC domain class transcription factor

TTAATATCTTCTTCCATTAAGCAAAAACCACCCGCTGAAAGCCGAACACTTTGATTAAACCCCAAGTCATTGTCCGTACT

GTTTCTCCTGTGTTTCTAATCCTATACAAATCCACCCAGATTCCGATTCTCCTTCATGAAAGATAGATGGCTGCCAATAG

AAACCGCTTCACCAGAGCAGCAGCAGGTGACCTGTCGATGCCAGTGGGATTCCGGTTCCGCCCTACAGAAGAAGAGCTGA

TCGACCATTACTTGCTGATGAAGCTCGAGGGTAAGGATTACCTTGTCAGCGACGTCATCCGTGAAATCAATATCTGCAAC

CACGAGCCAAGGGACTTGCCTGGGTTTTCACTCTTAAAGTCGGATGATCAAGAATGGTACTTCTTCAGCACTTACAAAAA

AAACAGCAGAAACCAGACCGAAAGGGCAACGAAGGAAGGCTTCTGGAAGATCACTGGTGCCCAAAAACCGGTCAAGACTC

AAGACAATAGAACTATCATCGGTAAAAAAAGGATTCTAACTTTTTACCGAGGTACTGTTCGTAATTCCCAAAGGACCAAC

TGGGGTATGCATGAGTATTACATCCCCCAAACCAATCCTAATGCTAATCAGAGGGATTTCGTTCTCTGTTACGTAAAGAA

GAAATCAGGTAAGAACACGGATGTTGCAACCGGTGATGAAGGTGAATCTAGTAATTACAACAATGCACATGACTTTGAAA

ATCAACTGCAACCAGCACGTAATATGCATGTTGAAGAGGAACGTACTCAGCCACCACCAAATCCGGACATTTTTCAGAGG

GAATTGGATAGTATGCTTGGACATACGCGTGGTAATAATGATTACCATGGAACGCAATCTGCACCTGGAGCTAATGAATC

GGATGGAGATTCAGAAGAGTTCGTAAATGGACTTTTTGGGGATGACCCGTATCAGGGTTACAGTGAAGAAATAACTCACA

ATAACGTCTTTAACGACTCCACTCTGCCACAGTCACCTAGGAAGGTGTATTTAAAGGATTGTGGAGTAAGCAGTGGCTCA

GCCACAGAAGTACAACATGCAGTTGATGAAGAGGGCCAGTCCCAGGGCGGGAAAGGTGTTCCGCTTCGTAACCGGATCTT

AATACAGGCTGCTTCCTCTACCAATGTATTACCTAAACCTCAGACTGATCGTCTTCAATCAGTCAGTGATGAAGATTCTG

GTGCATCAAGACCCATTAAAAAGAGTGCTTCCTCTGTGGATGTAGATGCAGATAGCCTTCAGATTAACTCTATTCAGTTA

GCCTCCGACGAATACTATAACAATGAGAGAACACGCAGAAGAACATATCCAACAAGTGCCCTCGAAGCACTAAGCAAACA

GAAAGAATTAAAACAGCAGCAAAGCAAGGCAAAAGAAGCTGCAGAGCTTCGAGCTGCTGTTGATTTTCCTCCGAAGTTGA

CATCTATAACAAAGTCTAACAAAGAAGCAGAGGTGGCTCAAGGTAATAATGCAGAAAAGGGTGTGAAGCAGACACAGAAC

ACAACAACCACTTGCAATTGGAAGGGCTGTTCTTTCATTTCATGGGAGACATCCCCCCCATTAACAAGTCCCCCATCAGA

ATACTTTTTCAACATGGTTCTAGGCATAATAATGTTCTGCTTTTTTGCTTGGCTAGTAGTTTTATACGGGCAGTGGTGCT

AGAGGGGCTCATTCACCAGAAAAATGGTTAGTTTTTGTTGTCTGTCACACGATGAGTAATGCCATACTTACTGGTTACTT

ATTGCTGCTCTAAAATGTGGGAGTACCAGCCACGGTTTGCAGTGATGAAGTGTTTATATTTGGTATAGCCTATAGAGTTT

GACTTTTTTCTGTTTCTACGGACCAGATGTAATGATGGATGGCTAGTCCACATAATATATTGTTTTTTGCTTAATCGTTT

TGGGAGCAGGAATTCGAACTTTTGG

>MdNAC58 [Leaf=Malus domestica] NAC domain class transcription factor

GGATCCAAATAATCCAGAAAAGTCGACAATAAAGTTTTTGGGGACCAGAAAACAAGTTCGTGTTCAATTCCCGAGGGCAG

TACTGATATAAATCTTACAAATCACTGACATTTTCTGCTCGGCTGTCCTTGTTTCTTCCCTGATATCTGTAACCTCCGTT

CAGCTCCCTCTATAGTTTGAAGTCGTGAGGAAGATTGACACACAACCGATCAGATCCGTGGACTGCAAATCTAAAGATTT

TTCTTTTCTGACCGAAGACCAAAAGCTGGACAATATGGAAACTTTTTCACACGTTCCCCCTGGTTTCCGGTTCCATCCGA

CGGATGAAGAACTTGTTGATTACTACCTCAGGAAAAAGGTTACTTCAAGAAGGATTGACCTCGATGTTATTAAAGATGTC

GACCTCTATAAAATTGAGCCATGGGATCTTCAAGAAATATGCAGAATAGGGACAGAAGAGCAAAACGATTGGTACTTTTT

CAGCCATAAAGATAAGAAGTATCCTACTGGAACTCGAACAAACAGAGCCACAGCTGCAGGGTTTTGGAAAGCCACAGGAA

GAGACAAGGCCATTTATTCAAAGCATGACTTAATTGGCATGAGGAAGACCTTAGTGTTTTATAAGGGTCGAGCCCCAAAT

GGTCAGAAGTCCGATTGGATTATGCATGAATACCGCCTTGAAACTGATGAAAATGGAACTCCGCAGGAAGAAGGATGGGT

TGTATGTAGAGTATTCAAGAAGAAAATTGCAAGCATGAGAAATAAGATGAGTGAACACGAGTCACCATGTTGGTACGATG

ACCAAGTCTCCTTCATGCCAGACTTGGACTCACCAGGGCACAACTCTAGCTCTATGAACATGAACATGGTGCCATACCAC

CAGCTTCCTAGTTATGCATGCAAGAAAGAGCTTGATCACTTGCCCTTCCAGGTTCCCCACGAGCACTTCTTCCAGCTCCC

TCTTCTCGGAAGCCCAAAACTACTTCAATCATCATCAGCCACAGCTGTGAGCAGCTCCAACTCCATGGCACCTCATGCAT

ATGGTATCGACATTAACCACGCTTGCACTTTTCAGCCATTAGATCAAGACCAAAACTTTCATGGTGTAGTCTATGGTAAC

AATAGTAATGATGATCATCAGCGAACAGTAGATCAACTGACTGATTGGCGAGCGCTGGACAAGTTTGTAGCTTCACAACT

TAGCCAGGATGACGCCTCAAAGGGAAATAGCTACTCATGATATTACATGCCCTGTTAATAAAATGTCTATATTATGTATT

AGACAAATATGGATTAATACTCAAACATTCGTAAGGAAGCGACACCATATGAAATTAATCATTTAGTAGTGTATGTGTGT

GTGTATATATATATATACATACTGATCATAGGATTTAAGTACATTAGGGATAATCGAGAGGATATTGATCTCAACATTTC

>MdNAC59 [Leaf=Malus domestica] NAC domain class transcription factor

ATTACTAGTACTGAGTGCTTGACGAACTAGCTTGCTTTAATTAGTTACGAACTAGCTAGCTTTAATTAGTTAGTTTCTAA

TCAGTTTATGGCAGTTGCAGAAACAACAACAACCTCATCATCATCAACAATGAGCCACGACGACTCCAACAAGGCCGACC

ATGATCATGAGCATGAGCATGATGAGTATGTTGATGAGCATGAGCACGACATGGTGATGCCGGGCTTTCGCTTCCACCCA

ACTGAGGAGGAACTTGTAGAGTTCTACCTTCGCCGTAAGGTTGAGGGCAAGCGCTTCAATGTTGAACTCATTACTTTTCT

CGATCTTTATCGCTATGACCCTTGGGAACTTCCAGCTATGGCAGCAATTGGGGAGAAGGAATGGTTCTTTTATGTGCCAA

GAGACCGCAAGTACCGCAACGGCGATAGGCCAAACCGGGTAACTACTTCTGGGTATTGGAAGGCAACTGGAGCCGACCGT

ATGATCCGAAGCGAAAACTTCCGCTCAATCGGCCTCAAGAAAACCCTAGTCTTTTACTCCGGGAAAGCTCCCAAAGGCAT

TCGAACTAGTTGGATCATGAACGAATATCGCTTGCCGCATCATGAAACTGAACGATATCAAAAGGGAGAAATATCACTAT

GCCGAGTATACAAGAGAGCCGGAGTTGAAGACCACCCATCACTCCCTCGTTGTCTCCCGGCATCCAGGGCATCCACATCT

AGATCATCAGTACTAGTGGCTCAATCGACGACCAGGTCAGACAATAAAAAGCAGCATACCAATAATATCAACACAGTTGA

TAGTGTGATTGAAAAACTCCAAGCTTTTAATGAAGGCCAATCGCAGCAGATTCATCATGATCATGAACATCGGATGGATC

AAAAGATGAACATCGAAACTGAAGGTAGCAGCAGAAATTCTGATGTGACAACAGTTTTGCGGCTTTCCAAGCATAAAGCG

TACCCTCAGGGCCACCACCAGCACCACAACATTGAGAGTAGTACCGCTGCCGCACCTGGACAACCGGGTGCTCCAACTCA

TCCTGTGGATCAGGAAGAAGGGATGGCTGCTTTCTCGCACCACTCAGCAAACTCAAAGCAATCTGGTGCTAATTGCTCTT

CGGTGATCTCGAGTACTGGCAATTCATGTGCAGCACTCTTCACGAGTGGTTCTTCTTCTGTTTTGTCTTCCAATATGAAT

GCGATTGATGATCTTCATAGACTTTTGAATTACCAACCTGTACACGTACAGCAGCAGGCTGCTTCCAATCATCACCAACA

TCAACAATATGTACATCATCATCTAGTGCAATACTATGATCATCATCATCATCATCCCCAACCCAGCAACCTTTTCTCAA

CAAATTTCCCTTCAGCGGCGGCAGCACTATCAACTATACAGCCACAGCCGCAGCAGCAGCTCCCCCCCAACGCACTTCCA

ACTGCTTTCTCTGACCGCCTATGGGACTGGAATCCAATATCAGAGCCAACCCGGCCGGACTACAACAATCTGTTCAAGTA

ATCCAATTATATCAGTACATATTTATAGTACTGATTACCATGGATTTTCTTACATATAGATCCATAATAGTTCCATCGAT

AAACTTCTTAATTAGTTATGCTATTAGCTAGCTACTTTAATGAATGATTTATCTAGCTAGCCAGCTAGCTTACCCCTTAA

TAAGTTCTAGGTCTAGTAATCTAATTGCTTAATGTTCTAGCACATTAAGCTTATATATACTTGATTTTTTCTTTTAATGT

TATGACTTATATATATAATATGTTTCATTGTTTCTCTTCAAAAAAAA

>MdNAC60 [Leaf=Malus domestica] NAC domain class transcription factor

TTCTTTCTTTCTTCTTCTCTGACTTGTCTCTCTCCCTCTTTCCTCTTCAA

TTCGATCGATCGGCTAGCTGCAGATTCATTCGTTAATTTTCTTTCTTTTC

TTCGGTTTGTAGTTGAAATTAATTGTTGGATTGAGAATACTTTGTATTCT

TGAACTTAGCTGGCTTGCTAGGCAGCCCTTATATAAAAAGAGAAATTGTC

GGCGTACATATACATATAACAAAAGAGGAGAGATGGATCCGTCGATTGAA

GCACAAACAGAAACAGGCATGGTGGTGAATAGAGGATTATCAGACGAGCT

CAAGGGGTTGCCTCCGGGTTTTCGGTTTCATCCCACGGATGAAGAAATCA

TAACTAGCTATCTCATAAAGAAGGTCATCAACAGCAGCTTCGAAGCTGTT

GCAATCGGCGAAGCTGATTTGAACAAGTGCGAACCTTGGGATTTGCCTAA

AGAAGCAAAGATGGGAGAGAAGGAGTGGTACTTCTTTTGCCAGAGAGACA

GGAAGTATCCGACGGGGATGAGGACGAATCGTGCGACGGAATCAGGGTAC

TGGAAGGCCACAGGAAAGGATAAAGAGATATTCAATAAGGTTAAAAAGCA

GCAACAGCAGGGCGGGAAAGGTGCAGGAGGAGGATGCCTGGTTGGGATGA

AGAAGACTCTTGTGTTCTACAGAGGGAGAGCCCCTAAAGGAGAGAAGACC

AACTGGGTCATGCATGAATACAGGCTTGATGGCAAAATCCCACTCTACAA

TAACCTCTCCAAGGCTGCAAAGGATGAATGGGTTGTGTGCAGAGTTTTCC

ACAAGAACATGGGGCTCAAAAGAACCCTTACCCCAACTCGGATGAACTCT

TTTGGGAATATTGGGGATCACGATCTCTTGGATTGTTCTTCCCTCCCACC

TCTTATGGATCCTCCTCTTAATACTTCCAACATCATGACCAACAAACATT

CTTCATGCTATGGAGACAATGACTTCAAGCCCGATTCCATTATCACAGCG

CCACACCCATCGGATTATCGTGGAATCAACACTAACAGTTACCTCAACTA

CGTTTCACTGGGGGGAGGCGGCCGCGGTGTTCAAGGCCTCAAGCAGAGTT

GCAACTTTCAGCTGCAGCCTAACAATAACTACAACAGTTACCAAGCCATT

AGTCTCAGTAACCCTTCCAACATGTTATATCCTCCACCTCTTCATCATAA

TCATCAGCATCATCACCAGTTTCAAAATCCTTCTAGTTTCCCAAATTTCC

AGCGAAATCAGATGATGAGCGATTCTGATTATTTTCAACAAGGGATGATG

AGGGGTACTCAAGGGCAAGGGTCGGTGTTCCAAACAAGTACTATTACTAG

TAGTGACCGTGATGACGATGATCAGGCCATTCTAAAAGCAATAGCTGCAG

ACAACAACAACAACAACAAGAGGAAGAATAATAATGAAACATCATCGTCT

GGCTTAGGAGGAGGAGGCTTAGACAGGCACTGCAAGGTCGAGCAGTTTTC

GTCCAACCAGTCTATGTTTAGCCTCTCCCAAGACACCGGATTGAGCACCG

ACATGAACACTACCGAGATATCATCATCATTTATTTCAAAGCAAGAATTG

GGCAGCACCAACAACTCATCCTATGATCAAGATCCATCTGTTGTCCCCCT

TTCCGACATTGAAGGCTTGTGGGGCTTTTGACATTCATTCAAAAACAAGT

CGCGCCGCCTTCTGCTAATTGGGATTATTAAGGGTTTAAATTTTTTCTTT

ATTTTGGTAGGTAGATTAACTAGTCAGAAATGGATGTTGTTGCATACACG

AATACTGTATAGTACTTTCATGGTTTCCGGGGCCATTTTAATTTTCATGA

TTATTGTGAACTAAACTCATGTAAATTTTCTTTTTTATTATTTAAATTTA

T

>MdNAC61 [Leaf=Malus domestica] NAC domain class transcription factor

TCGGTGTCAGCGTGGAAGCAGAGTCGAAGCGGAGAGAGAGAGAGAGAGCAAGTCTGCTACGGTCTCCGAAGACGGAAGCT

TTGCCTCTGAGTTGGGCTTCTTTATCGTCGCCGCTCATCCATGGCTGGGCCATCATGGTTGTTTGACAAAAATAGAATCG

CAACAAAAATCAGGAGTGCATCTGGAAGCTATGATTCTGTTGGAATTAAATGGCAAAGCAACCCAACTAAATCTTGTCCA

AAATGCCAATATACGATGGACAATAGTGATGTTGCTCAAGAGTGGCCGGGTTTACCCAAGGGTGTAAAATTTGATCCAAC

CGATCAAGAGATTATGTGGCACTTACTAGCAAAATCTGGTGTAGGTGATCAAAAACCCCATCCTTTTATTGATGAGTTTA

TTCTAACTGTCGATGACGACGAAGGAATCTGTTGTTCCCATCCTAGGAAACTACCTAATGTTAAGCTAGATGGAAGTCCA

TCCCACTTCTTTCATAGAGCAATTAAGGCTTACAATACTGGAACACGAAAGCGGCGAAAGATACATGATGGTGATGGTGA

TGGTGATGTCCGCTGGCACAAGACTGGAAGGACTAAACCAGTGATCTTGGATGGAGTACAAAGAGGGTGCAAGAAGATTA

TGGTTCTATACATGAGCACGGTCAGAGGAGGAAAACCTGTGAAAACCAATTGGGTTATGCATCAATATCACCTAGGCATT

GAGGAGGATGAGAAGGATGGAGAGTATGTTATATCAAAAATATATTACCAGCAGCAGGAAGCCAAGCAAGCTGATAAAAC

TGATCAAGATGTTCCTGAAAGTATTGATGCGGTGATCACAAAGGTAGATCCAGTCACTCCCAAGTCGGCTACTCCTGAAC

CTCCACGCGCCGAACGAAAGCTTGGTGATTTTCATTTGGGACTGGATACTCCTGCTCCGTCCAGAGATCCCTTTCTTCAG

TATCGTGAGGAAGACGGTGCTGAAGATGAAGTGCGTCCTGATGTAGACTCTGCTTTGGATGAAGTTCATCCCGAGGTGAA

CCGTGCTGAAGATGTTCGCCCTGAATCTGAAATTCCTGACGATTATGATCAACACAAGGTAGAAAACCAAGCCGATGAAG

TAATTAATAACACTGAAAACATTGATCATGCTCAGGAAGATCCAAAATGGTGGGACAATGAGTCACAGAACCTTCTGGAT

TCGCAACAACTTGTGGAAGGGTTGTCTCTGTGTGATGAGCTCCTTCAGAGCCAATCTCCAAATAGGGGTGGGCATGAAAA

TGGTGAACAAGCACATGTCAAACCCCGTCTTGCTGATTACGCTAAACTAGGACCTGAGGATTTAAAGAAGGATCTAGAGG

CATGCCAAAATATTGTCCTTGATCCTGCTAATATTATGGACCTTGATACACCTCCTGATTTCCGACTCAGCCAGCTGGAA

TTTGGGTCACAGGAGAGTTTCCTTTCTTGGGGTGGCAAGGTGGCTGACTGAACATGGTTTGGGATTTCTGTGTTGGTTAT

GGTCAATGGTGGTAAATATTTTCTTGCTACGTTTTGGTTAAACTATGCCTATCAAATCAGCTTGGCGTGATATCTAACCT

TATGGTAAAGAACTGTTTTTTGTAACTGTAATTCTCTGATCGTTTGTGTTCAAACATCGATCGAACTTACTCTTTTTGCT

CTCTTTTAAGCATCAAGCTAAAATGCAGTTATGTTATCTGGTTTGCTTTTG

>MdNAC62 [Leaf=Malus domestica] NAC domain class transcription factor

GCTTTAGCTTTTTCAGTTCATTGGGATTTTCTCGGACATGGAGAGGATTA

ATTTTGTGAAGAATGGTGTGCTGAGATTGCCTCCCGGTTTTCGATTCCAC

CCGACGGACGAGGAGCTTGTTCTACAGTACTTGCGGCGCAAGGTTTACTC

CTGCCCCTTGCCTGCCTCCATCATCCCGGAGGTTGATGTCTGTAAGGCCG

ACCCTTGGGATTTGCCAGGTGATTGTGCGCAAGAGAGGTACTTCTTCAGC

ACTAGGGAGGCCAAGTACCCCAATGGGAACCGATCGAACAGAGCGACGAG

CTCTGGTTACTGGAAGGCGACCGGATTGGACAAGCAGATTGTGACTTGCA

GGGGCGGCCAAGTCGTGGGGATGAAGAAAACTCTGGTTTTTTATAGAGGA

AAGCCTCCCCATGGCACTAGGACCGATTGGATTATGCACGAATACCGCTT

TGTTTTGCCCGAAAATCCGGCCTCCATTGCCCCACCGGAAAAGAATGCAA

CCCAAAGCCCTGTGGTGCCAATGGACGACTGGGTTCTTTGCCGCATATTT

TTTAAGAAAAGGGGAGGCAAAAATGAGGAGCAGCAAGTTCAACAGCCCTC

CTGCGATGTTCGAAAACCCAAGAACTCGAGGCCTGTTTTCTACGATTTCA

TGACGAAAGACAGGGCAAATTTGAGCCTTGCACCTTGTTCTTCCTCCTCA

GGGTCGAGTGGAATCACAGATGTGGTTTCTAGTGAGCAAGTAGACAATGA

TCACGAAGAAAGCAGTAGTTCCAATAGTTTAGGTTTAATTAGAAGAAAAC

AGTAGCAGTTTAAATTAATTAATTTAAAATCCGCTTAAATTAGTTCGGTT

ATGTTCCAGTAATTGGGCTCGTTGCTCTGTCAGAATAAACATCTGAGAAA

AATCCAAGTTTAGGAAGAAAATCGGCAAGTAACTTGTAGTCGGAAATTTG

GAGAGTAATGGATGGTTTTTATAATTTATGTAATCAAATCCCACATTTTA

TAATATCTGATGTATTTACATTCTAAAAAAAAA

>MdNAC63 [Leaf=Malus domestica] NAC domain class transcription factor

ATTTCAAACTCTTCTTCCCTTTGTTTAATTTACAGTTTGGTTGTTCGCTGGTTCGTTCATTTTGCGACAACTGATAACAC

AAGCTCTCCTCCTCCAACTCTTAATTTCTTTCATGTACATGCTTTCATTCCTAGCATCTTTCTTCATCAATATTTGGTTC

AATTTAGGTAGCTAGGTATCTGTGGCTAGCAAGGAAATATTTGGTTGATTGAGGATGAAAAACCCAGAATCAAGCCTGCC

ACCAGGATTTAGGTTTCACCCTACAGATGAAGAGCTCATCCTTCACTACCTTGTAAAGAAGGTGGCCTGCACTCCCTTAC

CTGTTTCCGTCATCGCTGAAGTTGATATCTACAAGTTTGATCCATGGCAATTGCCAGCCAAAGCTGCGTTTGGTGAGAAA

GAATGGTACTTCTTCAGTCCAAGAGATCGCAAGTATCCGAATGGTGCGAGGCCAAACAGGGCTGCCGCATCAGGGTATTG

GAAGGCAACTGGAACAGATAAGACGATTGTGGCACCATCAGGAGGGCGGCAGAATGTTGGTGTGAAGAAGGCTCTTGTGT

TCTACAAGGGGAGACCCCCCAAGGGAATCAAGACCAACTGGATCATGCATGAATACCGCCTTCCAGAAAGCCCAAATAAC

TACACCACCACCACCAACCACCACAGAGCCGTGAAGCTCAATGATTTATCCATGAGGTTGGATGATTGGGTTCTTTGTCG

AATCTACAAGAAGTCGAATGTCGTGACCTCAGCAGCGGCACTGCCAAATGATCAAGACCAAGACGTTCAAGGAGAAGAAG

ATTTCCTCCACGATATCCTTTTACCAAGCTTGAAAAGTCCCTTTCCCAGCCTTGATCACAACATGATGAGTACTAGTACT

ACGAGCAGTCTCAAGCCTCAGAAATCTTGTTCCTTCTCCAACTTACTAGACGCCATGGACTACTCATTACTCACTAGTAT

TCTGGCGGATGGCCAGTACTATATCCCAACTGGAACTGGACTTCAATCAACTACTCCTAATAAGTTTAGTTATAGCTGTA

CAGGGAGTACTAGTACTGGATTAGACCCGCAACCGCTGTTCAATACCAATATCAGCAGTAACAGAAGTCATTTGTTTCAA

ACTCTCCCTCAGTACAACTGTTTGAACTTGCCATCATCAGCCCCAAACACGGAAAACCGGCTAAAGCGCCAGCATTCCAT

CACAGATGATCGTGGCATGTTATTATATCCATCGAAGAAAGACGTAAATTCCAATTGCAGTTTCACTAACACCGCCAATT

CATTCTTGAATCAGCAATTACTTCTGAGTAATCCTCGTTTTCATTTTCAAGAATAAAACCATATAAAAGAAAGAAGGAAA

GAAAAGAAAAATTACGGAAGGTCGGACCTGCCAAGCCTTGGTCAAGCTGTTTCGTATGGATCATAAGTTCATAACAAAAG

ATGGCAAAAATTAAGTACTGAAAGGAGTTTGATTATTTTTATATACAATCTGGCAATCTGGAATGGCATGCATGATGCAT

GGGAGATTGACCCTCGAAAACAATACAATGTTAATTGACATGGGTACAATTGGCAAATCAGATTTACTACTTGGTATGGA

TTATGAACATGAGAATTGATCTTGAGTTCTTTTTTCACGAGCGAAGGATATCTTCCCAATTAATGTAATTACAATTCCC

>MdNAC64 [Leaf=Malus domestica] NAC domain class transcription factor

TTTTTACCCAAATTTCCGAGCCATAAAGTATTCGTCTTTGCTCTGAACAAGCAGCAGGGTTGCGAAACTATTTAATTTTT

CTGCGAGCTAGGGTTTTTGAGAAATTGGGAAATTTGAGGAAGATCAGTGGTCGGAGATGGCGGAGCTGTCGATGGAATCG

CTGCCGTTGGGGTTCCGGTTCAGGCCCACGGACGAGGAGCTCATCAACCACTACCTGCGGCTGAAGATCAATGGCCGTGA

TTCTGAGGTCCGAGTCATCCCGGAAATCGACGTTTGCAGATGGGAGCCTTGGGATTTGCCAAAATTATCAGTGATAAAGT

CAGATGATCAAGAGTGGTTCTTCTTTTGCCCCCGGGATAGGAAGTACCCAAATGGCCATCGGTCAAATAGAGCCACTGAT

GCTGGATACTGGAAGGCCACAGGGAAGGACCGGACCATAAAGTCCCGTCAGTGCAAGTCTGCTTCTAACAGCAATGGCCA

AGTTGGGATGAAGAAGACGTTGGTTTTCTACAGAGGTCGTGCCCCTAAGGGGGAGCGCACCAGCTGGATCATGCACGAGT

ACCGTGCCACTCAAAAGGAACTCGATGGCACTGGTCCGGGCCAGGGTGCCTTTGTTCTCTGTCGCTTGTTCCATAAACCA

GAAGAGAAGGCTGATGTACCAAAGTATGACGAAGTTGAACAAACTGGTTTATCTCCTACCACAACTAAGTCATCTCCTGA

TGAGTCCTCAGATATAATTCAGGAAACAGCTACATCAGAGATCCAAGGTGAAAATCAGGCAGAAGGTATCATGAGGTGGT

TGACTGATAATTCTGACAACGTGACCCCTGATGCTCTCCCACCGCTACCTGACATTTTTATGGCCTCTGATGTGGAAGAT

CAAGGAGCAGTAGAAACGGGGATCCAGGGCCATCTGACAATAGAAGAAAATCCTGCATTCTATGAGTCTTTGGGCGGCCC

GATTGATTGCAAAGTATTCACACCGTTGCATTCGCAGATTAATGCAGAGCTGGAACATTATGTGGGTTCACCTTTTACCA

GTGACTTTGGCAATTATGATAATGTATTGCATTTTCAAGACGGCACATGTGAACGGGATGTACCCCTCAAGGAGTTGTTT

GATGAGTTCAGCAATAGCCATTATGGGAGCTCCTGTGAGGAGTCAACTAGTCAGAAGAACTTGGTCGTTGGAAATGAGAC

TTATCTATCTGGTCATGCATGCACCACGCTGCCAGGAAACTCATGTTTCAATGGTGCATGGGGTAACACAGACGCCAAAA

TCGCCCAGAATGATTTGCAAAGGAGAGCATCTGGCTTGTACAATGAGCAATTTGGTTCCGATGATTTGCAGAACACCTCA

TTTGGTTATTGGCAAGCTAAAGCTCAAGCATCATTTGATGACCGAAAACCTAGAATGGGAAATATGACAGACAGTTATTT

ATCTCAATGCTCATTTGCTGAGCAGTTTCCTGTGAGTTCAGTCAATGATGTGTCCAATAGTTTGCATGGATCTACCGGTT

GGAAGAATCTTGATAACCACAGCGGTGATGATGTTGGTGGAACTGGAATCAAGATTAGGGCTCGACATCCTCAACTACAA

CCGAAAATAGATAGCTTTGTAGATCAGGGCTCTGCTGCAAGAAGACTTCATTTATTAGTGGACCATTCAGTTGGGTCAAT

TAACCATGGCTTTGATCCTAAATTTTAGAGGGATGATGGCTGGTACCAGTGTCCTCGACGACGGTGATGGGGTGACGGCG

GTCACGACGGCGGGGGTTGCTGTCGAAGAGGAGAGAATCGACGACAAGGGTGATGGCCTGGGTTCTCTGGGATGTTGTCG

AGGAGGATGATGGGTGGTGCCGTTGTATTCGCCGAGGAGAATCGACGGTTGGTGAGGGGATTGGAGTCGAGGGAAGGACG

ATTGGAGTCGAGGGAAGGGAACTGTCGAGGGACTGAGGTGAGGTCTGAGAGTTTGAGAGAGTGAGAGTGAGAGTGAGA

>MdNAC65 [Leaf=Malus domestica] NAC domain class transcription factor

CCGGCATTCAGTCTGCAGCAGTGTTGTTCTCTCCGTTTGCGCTTTCGTCTTCGTTCCTGCCTCTCGATCCCCCTCTCTCT

CCTCCTTCCGGTCTTCTTTTTGCACCTGATGTCTTTTGATCTCAACCTGAAACTTTCATCAGTTATTAGAGCATTTTGTT

TTATGAGCCGAGGCCGGCTGTGGAGGTCGCGAAGGATATTGTTCCGAGACTGTACCTAGCCCTCCTTTTCCCGGGGGGAC

AGGGGGGAGTGTTGTTTGATAATAGTCTTTGTTCTGTAATCAAGAGTTCAGCTACTTGGTTCTTTCTGTCAGGGAATATT

GGTAGGAAGAGGGGTTGTAATCAGGTAGTTGAACTCTAATTTTTAATTTTGTGATGGGGAAAATGTTTAAGGCTCCTGGG

TTTCGGTTTCAACCAACCGATGTTGAGCTTCTAAAATACTTTTTGAAGCGGAAGTTAATAGGGAAAAGGCTCCATGTTAA

AGTCATCTCAGAGGTTGACGTTTACAAGTATGATCCTTGGGATCTTCCAGACAAATCTGGCTGGGACAGTGGAGATCTGA

AGTGGTATTTCTTTTGTCCGAGAGAAAAGAAATATGCACGTGGGAATAGAATTCAACGTGCTACTGTAGGTGGTTACTGG

AAGACCACTGGAAAGGATAGGTCTGTTCTTTGCAGTGGTGCAGTTGTCGGGTGGATAAAAACTTTAATTTTTCATACCGG

TCGAGCCCCACATGGAGATCGAACGGATTGGGTTATGCACGAGTATAGGCTTGAAGATCAGGGCCTGGCTGATAGGGGTG

TACCTCTGGACTCATACGTTCTCTGTATGATTTTTCAAAAAGAAGGGCTAGGGCCAAGAAATGGTGCGCAATATGGTAAA

CCTTTTAATGAGGAAGACTGGAGTGATGACGAGGTTGCTGAAGGATTTGAAGATGCAAACACACCTGGCCCATATCTGGG

GCTGCTGTGCAACGAAAATAGTCCAATTGCTAATAATACGCATTCTCTTGAGGATATTGGCATAGGTCCTCCATCTGGAT

CATGCATATCTGATATTTTACCGCAGTCTTGCAAAGTTCTTCAACCAGTTTCCAGTAACTATGTTACGATGGAGAAGTCT

CATGCTTCCCATGGTGATGACATCCTGCCAACTTTTGATTACTCCAGAGAAGAAAACACTTTCTTTATGAGTGAGAATGG

GAAAAGTATGGCTCAAATCCATTCAGAAGCAGTTCCTAATGCAAGCATGCCCAAGCTAAATCATGTGCTGCCAAGCAACC

ATAATGGTTTTGCTTCTACTAGTGCATTCTTTTCTGAGAGAATAGGCATTGGTCCATCTGAATCATTCATGTCTGATATT

GCGCCACCACATTCCAATGCTCTTCAACCGGAAACTGGTAATTATGTTACAACAGAGAAGGCTCATATGTCTGATGGTGA

TATCCTTTCGATGTTTGATTACTTTCCTGAAGAAAGCACTCTTTACATGTATGGAAATGATAAAATTGAGACTGGAGGTT

GTTTAGAAACAGTCCTGAATGCAAACATTCCTGAGCCAGGCCAAGTGCTTGCGAGCAACTATAATACTTTCATCTCTACT

AATACCCAGTCTTCTGAGGTTATAGGCATTGGTCCTTCATTTCAATCATGCGGAATTGATGTTTTACCACCTTCTTGGGA

GGTTCTCCAACCGGTCTCCTGTAATTATGTTGCAATGGAGAAGACTCCTGCTTCTAATGGTGATGACATCCTGCCAATGT

TGGATTGCTTTGCAGAAGAAAGCACTTTTCTTATGAATGAGAATCACAATAATGAGGAACTGGATAATTTCATTGATTTT

GGCAACAACGCTCAAGCTATACCTCATCTGAATACAAGCAATATGTATGAAAATTTAGAAGACTTGGGCAACAACCTAGA

CGGATACAATTTCTCTGGCGTGCATGATGATTCTGCGAATCATTTTTTGGAGCTAGGTGACCTTGACCAACCAATGAATG

GCGATAATTCTGTGTAATTTCCTCTAGTGGAAGGGATCTATGGGTTTGAATTTATCAGTGAAGAGGCAGAGGCTCCAGAT

CGCATATTTGTGAGTGAAGTGGTTCATTGACTTCCTCCATATTATTATTGTCAATAATGATCAATTGGGTGCTGTGATCT

CCCTTTTTATTTGACTCTCCATATTATCTGTTGGTAATTTGATAATAACTGTTTGTTGCAAAACTGAGCGCAGGCGTTCT

AGAATTCATACAGCCGATCCCATTTAGTGGGATAAGGGTTTGTTGCTTTGTTGCAAAGACAGGAGTGTAAGTTTTACAAT

TCAGTATTATGTTATCATTTTCTTATAAAAAAA

>MdNAC67 [Leaf=Malus domestica] NAC domain class transcription factor

GAATTGGGTCACTTTGTTTTTAAAGACATACTTCACTTGCTCTGCTTATTCCGCTCTGTTCTCTGAAGGCAAAAAAAACA

TCTGATAATCCGTTTCCAAAACATTTGAAGGAGAGGAGGAGGGATGAGAAACATCAGCAGCAGCAGCAGCACCAACACCA

CCACAACCACCGCACAATCGGTGCCGGACATGGTGGCTGCGATCAGGTTCCATCCCACTGAAGATGAAAAGGTCGACTTC

TTGAGCAAGAAGATGAAAAGTCACAACTCTCAAGCCTGCCTCTTCCTCCCTTTCATCGATGTCCGCAAGTTCGAGCCTTG

GGAATTGCCTGAGCACATGTTCCCTGATTCTCCTCATCATGCTAAGGCGTGGTACTCCTTCAGCCCGTGTGGTTACAAAT

CCATCAATAGTCGTCGCTTCAATAGGGAAACAAAGAAAGGCTCTTGGAAGATGAACAGCAAGCAACGGGATGTGGGCTCG

AAATATTTCACTTGCAAAAAGAGGACATTAACCTTCCAAATAAAGACCTTCCAGGAAGGTAGTAAGTCTAAATCCGTTCC

GACCGGCTGGAAAATGCACGAGTACATTTGCATTAAACCTAAACGGGGTTCTAGTCCTCATCAGGAGAGGGATCAGCAAA

GGGACCTTGTTCTCTGCGTCCTGAAGTATAAGCCAGTTGATTCTAAGTCTAATACGGGTACTTCGATCCGTGGTGATTTT

GCTGATAGTACTGGTAATCGAGGCTACATTGCATCTAGTCCCGGAGATGATGAAGCTGCTACTGCTGCTGCAATGAATCA

AGATGATGGTGAACCTGGTGGCGGCAGCAGCTCATCTGATTTTATTAATAAATCTCTATGCGACATGATTCAAGAGTGGC

AGTCATGTGCTCCGGCAGTAGAATGTCTGAATTCATTCCTTCCTCTTCATGAACAACCACAGCTTGGACATTCTCTTGAT

ATCTGTAGTCATGAATTGCTTGATCCTGGTACTGGTGGCTGCATCACCTATAGTTCTGATAACCAAGCTGCTGCAACGAA

TCATATTATTCCCGACACAGAAGAAATTCTTGCATCCAAACAGCTAGAAGGAAGTGCTGTGCTTGGCAGTGGCAATCATG

ATGATGGTGAACCTTGTAGCGGTATTTCATCTGATTTTAATGACCATGCAGTAGATGATATGCAGGAGTTTGATCAGCTA

GACAAACTTTTGGATTTGCCCTTATCTCCTCCTGAGCTGTCTCAACCACACCAGCTGGAAAATGTTCCGGGCGTCCATAG

TGGCAAATTCAGTAGCTGGCCATCTCCAATTGGGGTTAATACTTCTTATGTTACTAATAAGAATAATATTGCAACCAATT

GCGAAAACGAACCAGTTATCAACGTCGCTTTTAATTCTTATAATCGAGCGACAGATGAAAGGATTTCCGAGGTTTATTCT

CAGGCAGAAGAAAATCTGAGTTTACCCTTTCAACCGTTTGATCAGCCTCAAAATTACACATGGCAGTCGCCAATGTTGTT

CTCAGAAGTGGGAGAGCTTCTGCATGCCAATAACTATATTGGATGCAACGAGTCGCAATTTCTAGATATGGAGGTTGCGA

CTCTTTTCCCACAAAGTTCTTGAATAACAG

>MdNAC68 [Leaf=Malus domestica] NAC domain class transcription factor

AGAAGGGAGGAGCATCGGATCAAGGCGGTGGAGTCGTTGGTCGTCCCTAAAGTCAAAAGACCTAATTGAGTTGTCATGTA

TCCGCAATCAGCTGCCCTGCCTGCTGATATCAGCTTACAGTGTACCGATGAGGAGCTCTGTGTGTCCTTAGAGAAAATAC

AGGACGGATCCCCGCTTCCTGGAAATGTGATTCGAGATGTAAATCCTTACCGATGCGCGCCCTCGAATTTACCCGATGGT

TTTTGGTACTTCATTCGCTCGAGTGAGAGCAACCCTACAAATATTGGGCAGTGGAGAGCCAAAGGGGAGGCCAGTAGAAT

ATTCTCAAACTCCTCCATCTATGGTTGGAGAGCTACACTTGAATTCTATCGAGGCCAAGCCCCTCATGAAACTAAAACCA

ATTGGGTGATGCAAGAATACTGGATAACTCAGAAGAAATCGTCGGAAAGCAGCAAATTGAAGATAAATGTGCAGGATGTC

GGGTCGCTTTGCAGAGTCTTTCTTAGGGATGATGAAACACCAAACTCCAATAAGCTTCAGGAATTGGTGAATTCTGATAG

GGCTATTCACTCAACACATTCAGTTGTTCCGCGTGTTGAGGATCGTGGTTCCACCAGCAAGCCTCGTGTTGAAAATGAGG

CAAGCAATGATCGCGAAACGGGAACATTGGTAGTTACAGAAAGGCTTCCAGTTCGAGATGATAATGTGCAAATTATGCAC

GAGAATGATTACCTTTCGAGAGGCGACTACTTGGAATTGCTGGATCTTGACACACCAGCATCTCATTCATTTAGTTCAGT

TTCGAGTTGCTTGACAATGTCCTCGGACGAATGCTTCGATTCTCTGGCATTGTTGGAAGAACTAGAGCCTAAAAGTAAGC

AAGAATTGGTGACGAACAACAATAAGTTGGTCGTGTTTGCATCCTCTTCAGGATCTTTCGACAAGTCTCGCGACAAAGAA

AGAATCAGAAATCGTTCTCCGAATCCTGGCTCATCTGTTTGTGCCAAGATTGTCACAGAAAATAGTTCAAAAAATGCGAG

CAGAAAACAAAAATCAGACGATAGGAATGAAGGGCCATCAAATTCTCAGAATGCTAATGTTGGTGGAACCAAAAAGCTTA

AGAGGAAGAAGTATTTCTGCTTCATGCCGTTTTAGGTCATCTGTTTTCATTCTACACGTGTGTATTTATGTATATTAACG

AATTTGAGTAGCAAATAATCAACATCATACTTAGACATTTTTTGTATTGTTCAAATGAATCATTTTGGAAAAAA

>MdNAC69 [Leaf=Malus domestica] NAC domain class transcription factor

TCGCCCGGAGCAGCTCTCTTCTTCCAGTTGGCTCTTCCACTTCCGCCCTCCCATCAGCGCAATCGCCGCCGATTCCCCCC

GTCCAATCTGCCTTCGCCAGTTCCCCCATCGCAAGGAGTGCAGCCAATGTCTTTTGATCTCAACAACGAGCATCCATCAA

TTATTCGATTGTTTTGTTTTATGAACCGAGGTAGGCTCTGGTGTTTGCGGAGGATCTTGTTCCGTCATTGGACTTGAGGA

GACGTGCTGCTTGGTTTCAAAAAACTATACTTTATTTTCTTCGCATCTTGTGAGGAATTTCCACATTGGGATTTAAGGTT

ATTGTTAGATAGTTGAGACGTGTATATTGTAAATGGGGAAAGGGAAATCATTGTTGCCTCCTGGTTTTCGGTTTTCTCCA

ACTGATGTAGAGCTTGTACAATATTATTTGAAGAGGAAAGTAATGGGGAAAAGACTCCCTTATAACTTTGTTGCAGAGGT

CGACATTCATAAGTATGCTCCTTGGGATCTTCCAGAAAAATCTAGCTGGCAAAGTGGAGATTTAAAATGGTACTTCTTTT

GTCCGACAGAAAGGAAGTACCCAACTGGGGCTAGAACGAAACGTACAACTGAATGTGGTTACTGGAAGGCCACAGGAAAT

GACAGATCTGTTCTTTACAATGGTGAAGTTGCGGGCAAGATAAAAACATTGGTTTTTCATACAGGTCGAGCTCCAAAAAG

AGACCGAACAGACTGGGTTATGCATGAGTATAGGCTTGAATCTAAGGACCTAGCTAATCGTGGTGTGCCTCAGGAATCGT

ATGTGCTCTGTACCATTTTTCAAAAAGAAGGGCCAGGGCCAAGAAATGGTGCACAATATGGTGCGCCCCTTATGGAGGAA

GACTGGAGTGATGATGAGGCTGAAAATTGTTCAGAAGCAGTCCCACATGCAAATATGCCTGTACCAAACCTTGTTCTGCC

GAGTGACTACAATAGTTCCATCACTACTAGCACGTACACCCATGATGGTATACGCATAGGTCCTTCATCTGAATCGTGTA

TATCAGATGTTGTACCACTTTCTTGCAATGTTTCCCAACTGGTTTCCAGCAATCATGCTACAGTGGAGGAGCCTCATACT

TCTGAGGATGATATCCTGTCAATGTTGGCTTGCTTCTCAGAAGAAAGCCCTTCATTATTTGAAGAAAACGAAAAAAATAA

GGAGGTTGGTAATGCCATTCCTGTTGGAAATGCTAGTGCAACGCCTCATCTCGTTATTAATGATATGTATGACAATTTAG

GAGATTTGGGCAAGGCAGCAAGAGTCGGTGAAGAAGGATGTAGTTTCTCCAGTTTGCCAAATTCTGTTTGTGCTCCGGGT

CAAATCCCACTAGGTGACCATGAGCAATATTTGGAGCTGGATGATCTTGGCGAACCATTGAATTACCGTGATTCTACATA

CACTCAGCCTCCTTCTATGTTCGGTCAGCCTCATGCTTCGCTGGGAGAGACATCTTTTCAGGGTGAAGACCAGTTGAACG

TGTTTGATAACGTGAACTTCTGAAGTTATTATTGCACTCCAGTCTTTCAAACCAAACTTGTTAAAACGGTTTCGCTAACT

TTCAGAAGTTCACCTTCTCAAACACAAACTTTGAGTTAATTCTGCACTTTCTTTTTCGTTGTTACGACAGAGCAGTAGTA

AATCCTAATTCACTAGTTGGGTGTTGGATTTGAATCGCGTCACCATGTTGTACAAAAATTGTGACATGGTATGACCAATG

ATTGCTTAGTAATTTAACATGTTATAACTAATTC

>MdNAC70 [Leaf=Malus domestica] NAC domain class transcription factor

TTGAACTAGCTACCAGCTTTGAGCTATTCCTATTTATTTGGGTAGCTGGTAGTAGCTAGTGTTATTAGGGTTAGGGTAGA

GGCAGGGAGATCGAGAGACAGATGCAATGGAGGATTTACCACCTGGATTTAGATTCTCTCCAACAGAGGAAGAGCTGATT

TCGTTTTACCTGCAGAACAAACTAGATGGGAGGAGTGAGGACTTGAACCGAGTTGTGGATCGAATCATACCCGTTGTCTA

CATTTATGAGTTTAATCCATGGGAACTCCCACAGGCTGCCGGAGAGGTGATCCATGGAGATCCAGAGCAGTGGTTCTTTT

TCATCCCAAGACAAGAAAGTGAAGCTCGAGGAGGGAGACCGAGACGACTCACAACAACTGGGTATTGGAAAGCAACAGGA

TCTCCAAGCACTGTTTACTCTTCCAATTCCAATCACTATCGTGCCATCGGCCTCAAAAGAACCATGGTTTTCTATACTGG

CAGAGCTCCCCATGGAAAGAAAACGGAGTGGAAGATGAATGAATACAAAGCCATTGAAATTCATGCAGATGATAATAATC

AACCATCAATGGCCGCCTCCTCATCAAACCCTAGTACTCCTTCTACTCCCACGTTAAGGCAAGAATTCAGCTTATGCCGA

GTATACAAGAAATCGAAATGCCTTAGGGCATTTGACAGGCGACCTCCAGGGATTGAGATCACAAGAAACCCTAACTTAAA

CATTCAAGCAGCTCCTGCTCAGACTGCAGATCATCTGGATCAGGGTTTGACAACATCGAATAGGAACCCTCAGAATATGG

AAGGGAGAACAAACTCAAGCTCACCGGAGAGTTCATCCTCAGGAGGACATGGCTCTCAATCCTCTCAACCAGAACAAAGT

GGGACTTTGCCAATGGCCGTTGATAATGAAGCTATTTGGGATTTGGATCAAATGATAAATTTTTTGCTTTAAGGGGTGGA

TAGGAAAAAATACTAAGGGGTATTACCACGTTATCCAAAATCGTTAAATACAATGTCTAGCTACTAAAAAAAA
