# Supplementary material for: Cloning, sequencing, and expression analysis of 32 NAC transcription factors (MdNAC) in apple
Source: PeerJ. 2020 May 6;8:e8249. doi: 10.7717/peerj.8249 (PMC7210808; doi:10.7717/peerj.8249)
Supplement: File S2 [file peerj-08-8249-s004.docx]

# Arabidopsis thaliana: NAP, AJ222713.1 (Guo and Gan 2006); ATAF1, NP_171677.1 (Lu, et al. 2007); ATAF2, NP_680161.1 (Delessert, et al. 2005); CUC1, BAB20598.1 (Takada, et al. 2001); CUC2, BAA19529.1 (Aida, et al. 1997); AtNAC1, AF198054 (Xie, et al. 2000); AtNAC2, AAO41710.1 (He, et al. 2005); AtNAC3, BAB20599.1 (Takada, et al. 2001); ANAC019, NP_175697.1, ANAC055, AAM61076.1, ANAC072, NP_567773 (Tran, et al. 2004); VND1, At2g18060; VND2, At4g36160; VND3, At5g66300; VND4, At1g12260; VND5, At1g62700; VND6, At5g62380; VND7, At1g71930; NST1, At2g46770; NST2, At3g61910; NST3, At1g32770 (Mitsuda et al., 2007); ANAC029/AtNAP, AT1G69490 (Shinozaki et al., 2014); ANAC092/ORE1, AT5G39610 (Carviel et al., 2009); ANAC016, AT1G34180 (Kim et al., 2013); ANAC102, AT5G63790 (Christianson et al., 2009); ANAC072/RD26, AT4G27410 (Fujita et al., 2004); NTL8, AT2G27300; NTL9, AT4G35580 (Kim et al., 2008; Seo et al., 2008; Seo, 2014; Yao et al., 2017 Yoon et al., 2008); FEZ, AT1G26870 (Willemsen et al., 2008); BRN1, AT1G33280; BRN2, AT1G03457; SMB, AT1G79580 (Bennett et al., 2010); ORE1, AT5G39610 (Qiu et al., 2015); ORS1, AT3G29035 (Wu et al., 2012). Oryza sativa: SNAC1, DQ394702.1 (Hu, et al. 2006); OsNAC3, BAA89797.1 (Kikuchi, et al. 2000); OsNAC4, AB028183 (Kikuchi, et al. 2000);OsNAC5, AK063399 (Takasaki, et al. 2010); OsNAC6, BAA89800.1 (Nakashima, et al. 2007); OsNAC19, AY596808 (Lin, et al. 2007); ONAC058, Os03g21060 (Zheng et al., 2009); ONAC122, Os11g03300; ONAC131, Os12g03040 (Sun et al., 2013); OsNAC10, AK069257 (Jeong et al., 2010). Solanum lycopersicum: SlNAM, NP_001234254.1 (Blein, et al. 2008); SlNAM1, EU670749.1 (Yang, et al. 2011); SlNAC1, EU670750.1 (Yang, et al. 2011); SlNAC2, SGN-U313171 (Uppalapati, et al. 2008); SlNAC3, SGN-U568609 (Han, et al. 2012); SlNAC4, KC453999 (Zhu et al., 2014). Solanum tuberosum: StNAC, CAC42087.1 (Collinge and Boller 2001). Triticum aestivum: TaNAC2, AY625683.1 (Mao, et al. 2012); TaNAC2a, HM027577.1 (Tang, et al. 2012) ；TaNAC4, GQ985329 (Xia, et al. 2010); TaNAC29, AML60267 (Huang, et al. 2015); TaNAC67, AHB32901 (Mao, et al. 2014); TaNAC69, DQ022842 (Xue, et al. 2011). *Hordeum vulgare*: HvNAC005, AK251058 (Christiansen et al., 2016); HvNAC6, AM500854 (Jensen et al., 2007). Brassica napus: BnNAC1-1 (AY245879.1), BnNAC5-1 (AY245881.1), BnNAC5-8 (AY245883.1), BnNAC5-11 (AY245884.1) (Hegedus, et al. 2003). Zea mays: ZmSNAC1, NP_001123932.1 (Lu, et al. 2012)。甘蔗 (Sugarcane): SsNAC23, AY742218.1 (Nogueira, et al. 2005). Petunia hybrida: NAM, X92205.2 (Souer, et al. 1996). Citrus sinensis: CitNAC, ABM67699.1 (Liu, et al. 2009); CsNAC, ABQ96643 (Fan et al., 2008). *Cicer arietinum*: CaNAC5, ACS94038 (Peng et al., 2009)。梅 (*Prunus mume*)：PmNAC, BAE48667 (Mita et al., 2006). *Gossypium hirsutum*: GhNAC2, ACI15342; GhNAC3, ACI15344 (Meng et al., 2009). *Bruguiera gymnorhiza*: BgNAC1, BAG15877 (Yamanaka et al., 2009). *Chrysanthemum morifolium*: DgNAC1, HQ317452 (Liu et al., 2011). *Mikania micrantha*: MmATAF1, ABZ89746 (Li et al., 2012). *Avicennia marina*: AmNAC1, ABS80935 (Ganesan et al., 2008). *Artemisia annua*: AaNAC1, KX082975 (Lv et al., 2016). *Ipomoea batatas*: IbNAC1,GQ280387 (Chen et al., 2016). *Picea sitchensis*: PaNAC03, ABK26029 (Dalman et al., 2017). *Arachis hypogaea*: AhNAC2, ACI42833 (Liu et al., 2011). *Glycine max*: GmNAC11, ACC66315; GmNAC20, ACC66314 (Hao et al., 2011). *Vitis amurensis*: VaNAC26, GSVIVT01019952001 (Fang et al., 2016). *Cucumis melo*: ETHQV6.3, MELO3C016540 (Ríos et al., 2017).

**References**

Aida M., Ishida T., Fukaki H., Fujisawa H., Tasaka M. (1997). Genes involved in organ separation in *Arabidopsis*: an analysis of the *cup*-*shaped cotyledon* mutant. The Plant Cell, 9: 841-857.

Blein T., Pulido A., Vialette-Guiraud A., Nikovics K., Morin H., Hay A., Johansen I. E., Tsiantis M., Laufs P. (2008). A conserved molecular framework for compound leaf development . Science, 322: 1835-1839.

Carviel J L, Al-Daoud F, Neumann M, et al. Forward and reverse genetics to identify genes involved in the age-related resistance response in *Arabidopsis thaliana*. Molecular Plant Pathology, 2009, 10(5):621-634.

Chen S P, Lin I W, Chen X, et al. Sweet potato NAC transcription factor, IbNAC1, up-regulates sporamin gene expression by binding the SWRE motif against mechanical wounding and herbivore attack. Plant Journal, 2016, 86(3):234.

Christianson J A, Wilson I W, Llewellyn D J, et al. The low-oxygen-induced NAC domain transcription factor ANAC102 affects viability of *Arabidopsis* seeds following low-oxygen treatment. Plant Physiology, 2009, 149(4):1724-38.

Christiansen M W, Matthewman C, Podzimska-Sroka D, et al. Barley plants over-expressing the NAC transcription factor gene HvNAC005 show stunting and delay in development combined with early senescence. Journal of Experimental Botany, 2016, 67(17):5259.

Collinge M., Boller T. (2001). Differential induction of two potato genes, *Stprx2* and *StNAC*, in response to infection by Phytophthora infestans and to wounding. Plant Molecular Biology, 46: 521-529.

Dalman K, Wind J J, Nemesiogorriz M, et al. Overexpression of PaNAC03, a stress induced NAC gene family transcription factor in Norway spruce leads to reduced flavonol biosynthesis and aberrant embryo developmen]. BMC Plant Biology, 2017, 17(1):6.

Delessert C., Kazan K., Wilson I. W., Straeten D. V. D., Manners J., Dennis E. S., Dolferus R. (2005). The transcription factor ATAF2 represses the expression of pathogenesis-related genes in *Arabidopsis*. The Plant Journal, 43: 745-757.

Fan J, Zheng-Guo L I, Gao X, et al. Cloning of *CsNAC* Gene from Navel Orange and Its Expression Analysis During the Storage of Fruits. Acta Horticulturae Sinica, 2008, 35(12):1803-1808.

Fang L, Su L, Sun X, et al (2016) Expression of *Vitis amurensis* NAC26 in *Arabidopsis* enhances drought tolerance by modulating jasmonic acid synthesis. J Exp Bot 67(9):2829-2845.

Fujita M, Fujita Y, Maruyama K, et al. A dehydration-induced NAC protein, RD26, is involved in a novel ABA-dependent stress-signaling pathway. Plant Journal, 2004, 39(6):863-76.

Ganesan G, Sankararamasubramanian H M, Narayanan J M, et al. Transcript level characterization of a cDNA encoding stress regulated NAC transcription factor in the mangrove plant Avicennia marina. Plant Physiology & Biochemistry, 2008, 46(10):928-934.

Guo Y., Gan S. (2006). AtNAP, a NAC family transcription factor, has an important role in leaf senescence. The Plant Journal, 46: 601-612.

Hao, Y-J. et al. (2011) Soybean NAC transcription factors promote abiotic stress tolerance and lateral root formation in transgenic plants. Plant J. 68, 302-313

Han Q., Zhang J., Li H., Luo Z., Ziaf K., Ouyang B., Wang T., Ye Z. (2012). Identification and expression pattern of one stress-responsive NAC gene from *Solanum lycopersicum*. Molecular Biology Reports, 39: 1713-1720.

He X. J., Mu R. L., Cao W. H., Zhang Z. G., Zhang J. S., Chen S. Y. (2005). AtNAC2, a transcription factor downstream of ethylene and auxin signaling pathways, is involved in salt stress response and lateral root development. The Plant Journal, 44: 903-916.

Hegedus D., Yu M., Baldwin D., Gruber M., Sharpe A., Parkin I., Whitwill S., Lydiate D. (2003). Molecular characterization of Brassica napus NAC domain transcriptional activators induced in response to biotic and abiotic stress. Plant Molecular Biology, 53: 383-397.

Hong, Y., Zhang, H., Huang, L., Li, D., Song, F. (2016). Overexpression of a stress-responsive nac transcription factor gene ONAC022 improves drought and salt tolerance in rice. *Frontiers in Plant Science,*7(e0116646), 4.

Hu H., Dai M., Yao J., Xiao B., Li X., Zhang Q., Xiong L. (2006). Overexpressing a NAM, ATAF, and CUC (NAC) transcription factor enhances drought resistance and salt tolerance in rice. Proceedings of the National Academy of Sciences, 103: 12987-12992.

Hu H., You J., Fang Y., Zhu X., Qi Z., Xiong L. (2008). Characterization of transcription factor gene *SNAC2* conferring cold and salt tolerance in rice. Plant Molecular Biology, 67: 169-181.

Huang Q, Yan W, Li B, et al. TaNAC29, a NAC transcription factor from wheat, enhances salt and drought tolerance in transgenic *Arabidopsis*. BMC Plant Biology, 2015, 15(1):268.

Jensen M K, Rung J H, Gregersen P L, et al. The HvNAC6 transcription factor: a positive regulator of penetration resistance in barley and *Arabidopsis*. Plant Molecular Biology, 2007, 65(1-2):137.

Jeong, J.S. et al. (2010) Root-specific expression of OsNAC10 improves drought tolerance and grain yield in rice under field drought onditions. Plant Physiol. 153, 185-197

Kikuchi K., Ueguchi-Tanaka M., Yoshida K., Nagato Y., Matsusoka M., Hirano H. Y. (2000). Molecular analysis of the *NAC* gene family in rice. Molecular and General Genetics MGG, 262: 1047-1051.

Kim Y S, Sakuraba Y, Han S H, et al. Mutation of the Arabidopsis NAC016 transcription factor delays leaf senescence. Plant & Cell Physiology, 2013, 54(10):1660-72.

Li, D.M., Wang, J.H., Peng, S.L., Zhu, G.F. and Lu, F.B. (2012) Molecular cloning and characterization of two novel NAC genes from *Mikania micrantha* (Asteraceae). Genet. Mol. Res. 11: 43834401.

Lin R M, Zhao X B, Wang M, et al. Rice gene OsNAC19 encodes a novel NAC-domain transcription factor and responds to infection by Magnaporthe grisea. Plant Science, 2007, 172(1):120-130.

Lui, X. et al. (2011) Improved drought and salt tolerance in transgenic *Arabidopsis* overexpressing a NAC transcriptional factor from *Arachis hypogea*. Biosci. Biotechnol. Biochem. 75, 443-450

Liu, Q.L., Xu, K.D., Zhao, L.J., Pan, Y.Z., Jiang, B.B. and Zhang, H.Q. (2011) Overexpression of a novel chrysanthemum NAC transcription factor gene enhances salt tolerance in tobacco. Biotechnol Lett. 33: 2073-2082.

Liu Y. Z., Baig M. N. R., Fan R., Ye J. L., Cao Y. C., Deng X. X. (2009). Identification and expression pattern of a novel NAM, ATAF, and CUC-like gene from *Citrus sinensis* Osbeck. Plant Molecular Biology Reporter, 27: 292-297.

Lu M., Ying S., Zhang D.-F., Shi Y.-S., Song Y.-C., Wang T.-Y., Li Y. (2012). A maize stress-responsive NAC transcription factor, ZmSNAC1, confers enhanced tolerance to dehydration in transgenic *Arabidopsis*. Plant Cell Reports, 31: 1701-1711.

Lu P. L., Chen N. Z., An R., Su Z., Qi B. S., Ren F., Chen J., Wang X. C. (2007). A novel drought-inducible gene, *ATAF1*, encodes a NAC family protein that negatively regulates the expression of stress-responsive genes in *Arabidopsis*. Plant Molecular Biology, 63: 289-305.

Lv Z, Wang S, Zhang F, et al. Overexpression of a novel NAC domain-containing transcription factor (AaNAC1) enhances the content of artemisinin and increases tolerance to drought and *Botrytis cinerea* in *Artemisia annu*a. Plant and Cell Physiology, 2016, 57(9):1961-1971.

Mao XG, Zhang HY, Qian XY, Li A, Zhao GY, Jing RL. TaNAC2, a NAC-type wheat transcription factor conferring enhanced multiple abiotic stress tolerances in *Arabidopsis*. J Exp Bot, 2012, 63(8): 2933-2946.

Mao, X., Chen, S., Li, A., Zhai, C., & Jing, R. (2014). Novel NAC transcription factor TaNAC67 confers enhanced multi-abiotic stress tolerances in *Arabidopsis*. *Plos One,* *9*(1), e84359.

Meng C, Cai C, Zhang T, et al. Characterization of six novel NAC genes and their responses to abiotic stresses in Gossypium hirsutum L.[J]. Plant Science, 2009, 176(3):352-359.

Mingku Zhu, Zongli Hu, Shuang Zhou, Lingling Wang, Tingting Dong, Yu Pan, Guoping Chen. Molecular characterization of six tissue-specific or stress-inducible genes of NAC transcription factor family in tomato (*Solanum lycopersicum*). Journal of Plant Growth Regulation, 2014, 33: 730-744.

Mitsuda N., Iwase A., Yamamoto H., Yoshida M., Seki M., Shinozaki K., Ohme-Takagi M. (2007). NAC transcription factors, NST1 and NST3, are key regulators of the formation of secondary walls in woody tissues of *Arabidopsis*. The Plant Cell, 19: 270-280.

Mita S, Nagai Y, Asai T. Isolation of cDNA clones corresponding to genes differentially expressed in pericarp of mume ( Prunus mume ) in response to ripening, ethylene and wounding signals[J]. Physiologia Plantarum, 2006, 128(3):531–545.

Mohammed N, Sharoni A M, Kouji S, et al. NAC transcription factor family genes are differentially expressed in rice during infections with Rice dwarf virus, Rice black-streaked dwarf virus, Rice grassy stunt virus, Rice ragged stunt virus, and Rice transitory yellowing virus. Frontiers in Plant Science, 2015, 6:676.

Nakashima K., Tran L. S. P., Van Nguyen D., Fujita M., Maruyama K., Todaka D., Ito Y., Hayashi N., Shinozaki K., Yamaguchi-Shinozaki K. (2007). Functional analysis of a NAC-type transcription factor OsNAC6 involved in abiotic and biotic stress-responsive gene expression in rice. The Plant Journal, 51: 617-630.

Nogueira F. T., Schlögl P. S., Camargo S. R., Fernandez J. H. (2005). SsNAC23, a member of the NAC domain protein family, is associated with cold, herbivory and water stress in sugarcane.Plant science, 169: 93-106.

Peng H, Cheng HY, Yu XW, Shi QH, Zhang H, Li JG, Ma H. Characterization of a chickpIea(Cicer arietinum L.)NAC family gene, CarNAC5, which is both developmentally-and stress regulated. Plant Physiol Bioehem, 2009, 47(11): 1037-1045.

Ríos, P., Argyris, J., Vegas, J., Leida, C., Kenigswald, M., & Tzuri, G., et al. (2017). *ETHQV6.3* is involved in melon climacteric fruit ripening and is encoded by a NAC domain transcription factor. *Plant Journal,* *91*(4): 671-683.

Seo PJ. 2014. Recent advances in plant membrane-bound transcription factor research: emphasis on intracellular movement. *Journal of Integrative Plant Biology* 56(4):334-342

Seo PJ, Kim S, Park C. 2008. Membrane-bound transcription factors in plants. Trends in Plant Science 13:550-556

Shinozaki Y, Tanaka T, Ogiwara I, et al. Expression of an AtNAP gene homolog in senescing morning glory (Ipomoea nil) petals of two cultivars with a different flower life span. Journal of Plant Physiology, 2014, 171(8):633-8.

Souer E., van Houwelingen A., Kloos D., Mol J., Koes R. (1996). The *No Apical Meristem* Gene of Petunia Is Required for Pattern Formation in Embryos and Flowers and Is Expressed at Meristem and Primordia Boundaries. Cell, 85: 159-170.

Sperotto R. A., Ricachenevsky F. K., Duarte G. L., Boff T., Lopes K. L., Sperb E. R., Grusak M. A., Fett J. P. (2009). Identification of up-regulated genes in flag leaves during rice grain filling and characterization of OsNAC5, a new ABA-dependent transcription factor. Planta, 230: 985-1002.

Sun, L. J., Zhang, H., Li, D., Huang, L., Hong, Y., Ding, X. S., et al. (2013). Functions of rice NAC transcriptional factors, ONAC122 and ONAC131, in defense responses against *Magnaporthe grisea*. *Plant Mol. Biol.* 81, 41-56.

Takada S., Hibara K., Ishida T., Tasaka M. (2001). The *CUP*-*SHAPED COTYLEDON1* gene of *Arabidopsis* regulates shoot apical meristem formation. Development, 128: 1127-1135.

Takasaki H., Maruyama K., Kidokoro S., Ito Y., Fujita Y., Shinozaki K., Yamaguchi-Shinozaki K., Nakashima K. (2010). The abiotic stress-responsive NAC-type transcription factor OsNAC5 regulates stress-inducible genes and stress tolerance in rice. Molecular Genetics and Genomics, 284: 173-183.

Tang Y, Liu M, Gao S, Zhang Z, Zhao X, et al. (2012) Molecular characterization of novel TaNAC genes in wheat and overexpression of TaNAC2a confers drought tolerance in tobacco. Physiol Plant 144:210-24.

Thirumalaikumar V P, Devkar V, Mehterov N, et al. NAC transcription factor JUNGBRUNNEN1 enhances drought tolerance in tomato. Plant Biotechnology Journal, 2017. https://doi.org/10.1111/pbi.12776

Tian H, Wang X, Guo H, et al. NTL8 regulates trichome formation in Arabidopsis by directly activating R3 MYB genes TRY and TCL1. Plant Physiology, 2017: pp.00510.2017.

Tran L. S. P., Nakashima K., Sakuma Y., Simpson S. D., Fujita Y., Maruyama K., Fujita M., Seki M., Shinozaki K., Yamaguchi-Shinozaki K. (2004). Isolation and functional analysis of *Arabidopsis* stress-inducible NAC transcription factors that bind to a drought-responsive cis-element in the early responsive to dehydration stress 1 promoter. The Plant Cell, 16: 2481-2498.

Uppalapati S. R., Ishiga Y., Wangdi T., Urbanczyk-Wochniak E., Ishiga T., Mysore K. S., Bender C. L. (2008). Pathogenicity of *Pseudomonas syringae* pv. tomato on tomato seedlings: phenotypic and gene expression analyses of the virulence function of coronatine. Molecular Plant-Microbe Interactions, 21: 383-395.

Xia N, Zhang G, Liu X, Deng L, Cai G, et al. (2010) Characterization of a novel wheat NAC transcription factor gene involved in defense

Xie Q., Frugis G., Colgan D., Chua N.-H. (2000). *Arabidopsis* NAC1 transduces auxin signal downstream of TIR1 to promote lateral root development. Genes & Development, 14: 3024-3036.

Xue G, Way H, Richardson T, Drenth J, Joyce P, et al. (2011) Overexpression of TaNAC69 leads to enhanced transcript levels of stress up-regulated genes and dehydration tolerance in bread wheat. Mol Plant 4:697-712.

Yamanaka T, Miyama M, Tada Y. Transcriptome Profiling of the Mangrove Plant and Identification of Salt Tolerance Genes by Functional Screening[J]. Bioscience Biotechnology and Biochemistry, 2009, 73(2):304.

Yang R., Deng C., Ouyang B., Ye Z. (2011). Molecular analysis of two salt-responsive NAC-family genes and their expression analysis in tomato. Molecular Biology Reports, 38: 857-863.

Yao S, Deng L, Zeng K. 2017. Genome-wide in silico identification of membrane-bound transcription factors in plant species. *PeerJ* 5:e4051.

Yoon H K, Kim S G, Kim S Y, et al. Regulation of leaf senescence by NTL9-mediated osmotic stress signaling in *Arabidopsis*. *Molecules & Cells*, 2008, 25(3):438.

Zheng, X. N., Zhen, B., Lu, G. J., and Han, B. (2009). Overexpression of a NAC transcription factor enhances rice drought and salt tolerance. *Biochem. Biophys. Res. Commun.* 379, 985-989.

Zhou J, Zhong R, Ye Z H. *Arabidopsis* NAC Domain Proteins, VND1 to VND5, Are Transcriptional Regulators of Secondary Wall Biosynthesis in Vessels. Plos One, 2014, 9(8):e105726.

Zhu M, Chen G, Zhang J, et al. The abiotic stress-responsive NAC-type transcription factor SlNAC4 regulates salt and drought tolerance and stress-related genes in tomato (*Solanum lycopersicum*). Plant Cell Reports, 2014, 33(11):1851-63.
