# Supplementary figures and images for: Cloning, sequencing, and expression analysis of 32 NAC transcription factors (MdNAC) in apple
Source: PeerJ. 2020 May 6;8:e8249. doi: 10.7717/peerj.8249 (PMC7210808; doi:10.7717/peerj.8249)

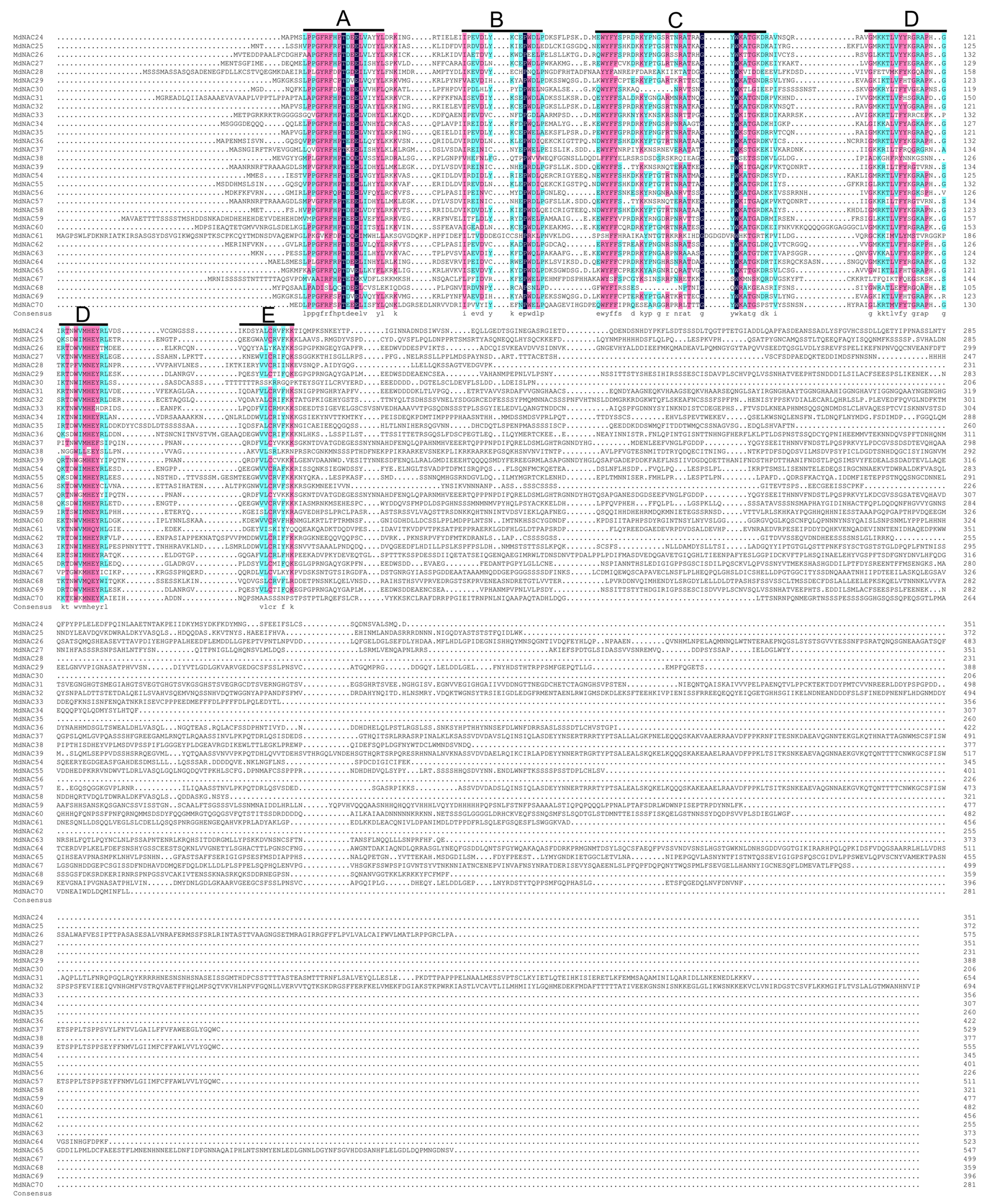

Supplement: File S4 [file peerj-08-8249-s006.png]
